# Investigating Transcriptional Dynamics Changes and Time-Dependent Marker Gene Expression in the Early Period After Skeletal Muscle Injury in Rats

**DOI:** 10.3389/fgene.2021.650874

**Published:** 2021-06-17

**Authors:** Kang Ren, Liangliang Wang, Liang Wang, Qiuxiang Du, Jie Cao, Qianqian Jin, Guoshuai An, Na Li, Lihong Dang, Yingjie Tian, Yingyuan Wang, Junhong Sun

**Affiliations:** ^1^School of Forensic Medicine, Shanxi Medical University, Jinzhong, China; ^2^Department of Basic Medicine, Changzhi Medical College, Changzhi, China

**Keywords:** time-series RNA-seq, gene expression and regulation landscape, time-related biomarkers, transcriptional dynamics, skeletal muscle injury

## Abstract

Following skeletal muscle injury (SMI), from post-injury reaction to repair consists of a complex series of dynamic changes. However, there is a paucity of research on detailed transcriptional dynamics and time-dependent marker gene expression in the early stages after SMI. In this study, skeletal muscle tissue in rats was taken at 4 to 48 h after injury for next-generation sequencing. We examined the transcriptional kinetics characteristics during above time periods after injury. STEM and maSigPro were used to screen time-correlated genes. Integrating 188 time-correlated genes with 161 genes in each time-related gene module by WGCNA, we finally identified 18 network-node regulatory genes after SMI. Histological staining analyses confirmed the mechanisms underlying changes in the tissue damage to repair process. Our research linked a variety of dynamic biological processes with specific time periods and provided insight into the characteristics of transcriptional dynamics, as well as screened time-related biological indicators with biological significance in the early stages after SMI.

## Introduction

Skeletal muscle injury (SMI), which has many possible etiologies, is commonly observed in daily life and trauma surgery practice (Zhu et al., 2007; Huard et al., 2016). Among many factors that may induce SMI, mechanical trauma- and sports-related injuries are common in both clinical and forensic practice (Best and Hunter, 2000). The skeletal muscle is a complex organ that is not easily regenerated *in situ* after traumatic injury, especially in cases of contusion injury (Sicherer et al., 2020). Therefore, mechanical trauma- and sports-induced SMI can induce dramatic and prolonged adverse effects on muscle functional capacity (Huard et al., 2002). Thus, in clinical settings as well as in many cases of intentional injuries, traffic accidents, insurance claims, and other emergencies, mechanical trauma-induced SMI can result in physical dysfunction. This has adverse effects on the activities of daily living and work capabilities of the injured parties and is related to wound age estimation in some criminal or civil cases (Grellner and Madea, 2007). Therefore, detailed analyses of the pathological processes occurring after SMI, gene expression changes, and damage repair mechanisms have far-reaching significance for clinical sports medicine and forensics.

Among many types of SMI, the general injury and repair mechanisms as well as causes are similar (Urso, 2013), particularly with regard to mechanical trauma-induced SMI. Wound response and healing are dynamic processes with degeneration, inflammation, regeneration, and fibrosis occurring sequentially (Li and Luo, 2019). To clarify the detailed pathological changes, biological processes, and possible mechanisms of the SMI repair process, there have been a number of studies at the levels of tissue morphology, cells (proliferation, apoptosis, and autophagy), proteins, nucleic acids, and molecular biology (Zhu et al., 2007; Zhao et al., 2009; Rybalko et al., 2015; Du et al., 2020; Sugasawa et al., 2020).

Although the changes in gene expression after SMI have been clarified, most studies performed to date were limited to distinguishing the type and degree of injury (Warren et al., 2007; Li and Luo, 2019; Yang et al., 2019). Some studies focused on changes in gene expression at various stages during the process of SMI repair (Sass et al., 2017), but these were based on open-source microarray data, and there were no clear time points indicating the divisions between repair stages. Although Xiao et al. studied gene expression changes in mice from days 1 to 21 after injury (Xiao et al., 2016), the early stage after injury, in which relatively rapid and diverse changes in gene expression and various biological processes have not been observed (Tidball, 2005; Stroncek and Reichert, 2008; Järvinen et al., 2013). In addition, most genetic time-series indicators come from the literature and theoretical studies, and to date, there have been no systematic screening studies and time-series algorithm analyses (Zhu et al., 2016; Gaballah et al., 2018). The maturity omics technologies, along with the adoption of big data and artificial intelligence approaches, have resulted in the widespread use of omics analyses combined with artificial intelligence algorithms, to study the processes of SMI repair (Camacho et al., 2018; Abdelmoez et al., 2020). Studies in this field have moved from a single feature to the integration of multiple aspects (Burnett et al., 2019).

In this study, we focused on the changes in gene expression and transcriptional dynamics in the early period after SMI in rats, to analyze the changes in biological processes and transcript profiles of the early period after injury. In this way, we analyzed transcriptional dynamics changes in the early period of SMI repair and screened time-dependent marker genes. This research provided a detailed transcriptome landscape of gene expression and biological processes changes in the early period of the SMI repair process, and identified marker genes that exhibited sequential variation in their expression with reference values for wound age estimation.

## Results

### Establishment the Early Skeletal Muscle Injury Model and Histomorphological Observation in Rats

**Figure 1:**
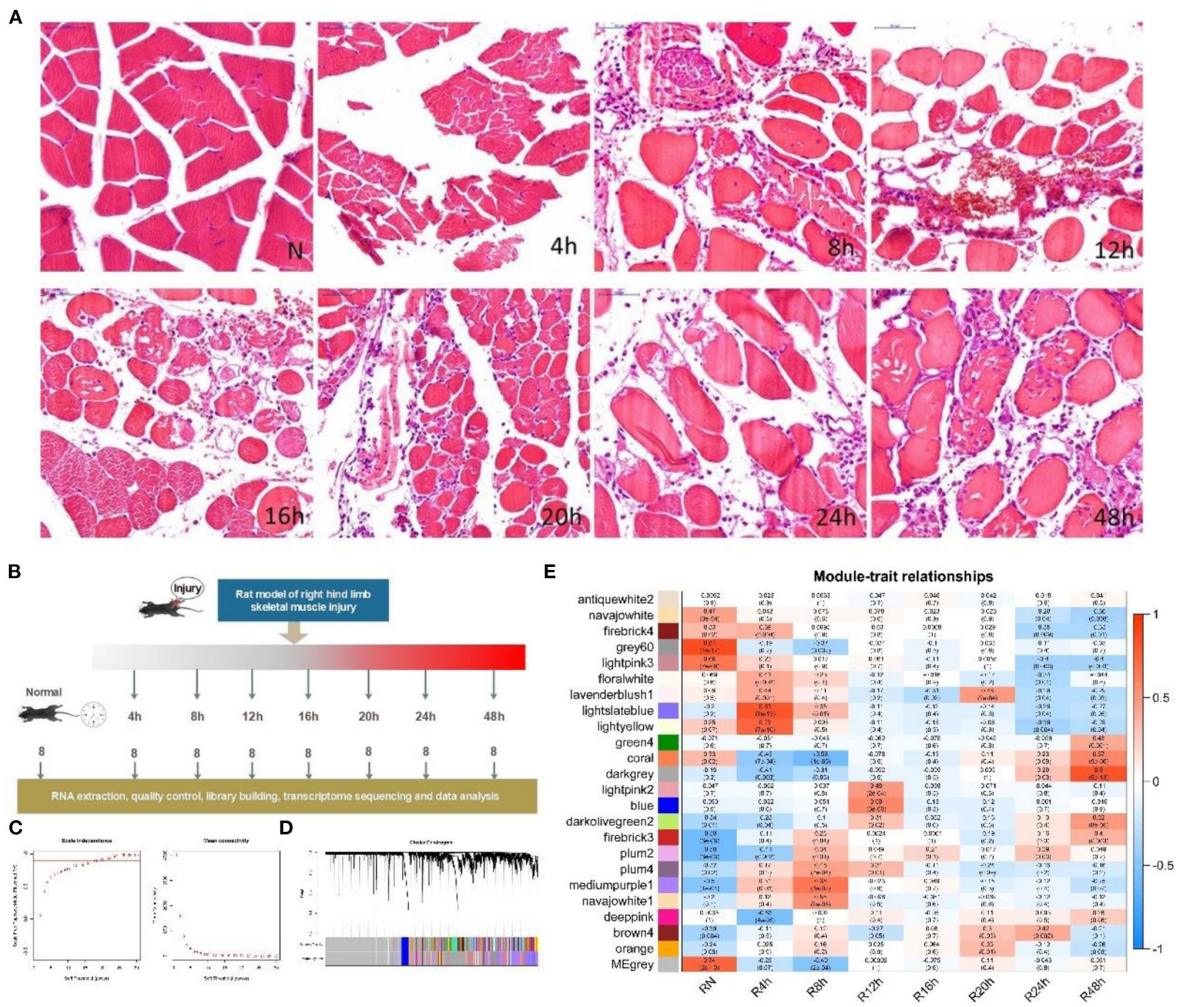
Experimental design, histological characteristics and gene co-expression module division according to WGCNA during the early period after SMI. **(A)** Animal model design. Sixty-four male Sprague Dawley rats were randomly divided into eight groups (*n* = 8 rats/group; seven experiment groups and one control group). Experimental rats underwent a SMI in the right hind limb and were monitored at 4, 8, 12, 16, 20, 24, and 48 h after injury. **(B)** H&E staining was performed to reveal the characteristics from injury to repair. The SMI repair process in the right hind limb in seven experimental groups (4, 8, 12, 16, 20, 24, and 48 h post-injury) and the normal group was histologically evaluated in transverse sections. Scale bar: 50 μm. **(C)** Relationship between soft power and network connectivity. Based on the scale-free network (y-axis, *R*^2^ = 0.94) with a soft threshold set to 22 and the mean connectivity value, the network exhibited negligible connectivity. The networks exhibited a scale-free network distribution. **(D)** Network cluster dendrogram. The network was divided into 24 co-expression modules each of which was assigned a color. The color gray was assigned to genes that did not cluster into a specific module. **(E)** Correlation between consensus modules and sample traits in each module. Red indicates a positive correlation, with a darker color indicating a stronger correlation. The top number in each cell indicates the correlation coefficient, and the bottom number indicates the significance of the correlation (i.e., the *p*-value).

First, we built an early state skeletal muscle injury animal model in 0–48 h using rats (see METHODS section). Then, we examined the injured rats in groups at seven time points after SMI (Figure 1B). To confirm the solid of animal model and the processes of muscle tissue injury to repair, we performed a preliminary histological analysis with hematoxylin and eosin (H&E) staining. H&E staining revealed the changes in muscle morphology after injury (Figure 1A). In comparison with the normal control group, the transverse sections of skeletal muscles exhibited breakage and the destruction of large numbers of muscle fibers at 4 h after injury. At 8 h after injury, interstitial blood vessels were congested with the infiltration of large numbers of inflammatory cells. At 12 h after injury, muscle fibers exhibited obvious hyperemia and edema, which reflected a strong inflammatory response at this time point. At 16 and 20 h after injury, the inflammation was reduced and the amount of fibrous tissue in the injured area gradually increased. At 24 and 48 h after injury, the numbers of fibroblasts increased, and muscle fibers were partially rebuilt. This was observed in longitudinal sections of muscle fibers at 4 and 48 h after injury (Supplementary Figure 1).

### Gene Co-expression Modules During the Early Period Post-SMI

To objectively describe and confirm the processes of pathological changes in the early period after SMI based on transcription profiles, we examined genes with obvious changes in expression, Gene Ontology (GO) terms, and pathways during the early phases of the post-injury response. In this study, we obtained raw next-generation sequencing data from 60 samples (in total 20,662 genes) (Supplementary Table 1). Then we subjected the standardized data (Supplementary Figure 2) to principal components analysis (PCA). After removing abnormal samples, the results of PCA indicated that the samples from each injury group were clearly discriminated from the normal samples and also that the groups at 4, 8, 24, and 48 h after injury were clearly distinct from one another. The groups at 12, 16, and 20 h after injury were not clearly differentiated (Supplementary Figure 3). These divisions were also reflected in the clustering correlation heat map (Supplementary Figure 4). Therefore, after removing genes with low expression, 15,901 common genes expressed in 53 samples (Table 1) were subjected to weighted gene co-expression network analysis (WGCNA) (Supplementary Table 2) (Zhang and Horvath, 2005). A soft power of 22 was selected to construct a scale-free network (Figure 1C). We then used dynamic tree cutting to divide the gene co-expression modules according to the common gene expression file. The clearest gene co-expression module distribution obtained at early post-SMI period using the Dynamic Merge tool included 24 modules comprising 23 meaningful modules and one undefined gray module (Figure 1D) (Zhang et al., 2019b).

**Table 1:** The number of samples, genes, selected GO terms, and KEGG pathways associated with the selected gene co-expression module in each group.

**Group Name**	**Normal**	**4 h**	**8 h**	**12 h**	**16 h**	**20 h**	**24 h**	**48 h**
No. of samples	6	6	7	6	8	6	8	6
Module Name	\	Lightslateblue	Mediumpurple1	Blue	\	Lavenderblush1	Brown4	Dark-gray
Co-expressed genes	\	1,118	1,946	455	\	11	102	2,028
Selected GO terms^*^	\	18	28	26	\	7	11	12
Selected KEGG pathways^*^	\	17	5	7	\	\	2	4
Name of the module	\	4	8	12	\	20	24	48

* *GO terms and KEGG pathways of interest were selected based on the threshold p-value (p < 0.01). “\” indicates that no module was selected for this group*.

To understand the correlations between each co-expression module and specific time points after injury as well as the potential biological significance of these modules, we used Spearman correlation analysis to calculate R values and *p*-values. Correlations with time and the biological significance of the 23 meaningful modules were visualized in a module-trait relationship heat map (Figure 1E). To analyze the expression of related genes in each meaningful module, we selected modules associated with positive time-point correlations (R > 0) (Figure 1E), setting *p* < 0.05 (Jin et al., 2019) as the threshold value. For each time point, we only considered the module with the highest R value.

Six co-expression modules (represented by different colors in Figure 1E, in this study we named the modules using the name of corresponding time point) were selected based on the results of WGCNA analysis (Table 1; gene annotation information for each selected module listed in Supplementary Table 2). However, we did not find an eligible module in 16 h group (Table 1 lists the correspondence between module color and time. The modules corresponding to time point in the following text are directly named by time). Finally, the most relevant module in each group, referred to as a highly correlated (HCr) module, was selected for gene expression analysis. Next, to confirm that the module division according to WGCNA and the selection of HCr modules based on gene co-expression profiles as outlined above (Table 1) were reasonable, we explored the relevance of gene co-expression in the HCr modules using heat maps of all genes that clustered in a given module. The results indicated that the modules identified by this analysis largely corresponded to the variation in gene expression at specific time points after SMI (Supplementary Figure 5). In addition, expression levels (corresponding to the feature vectors) (Zhang and Horvath, 2005) in the module of ***eigengene*** (represented by sparse loads of the most important feature vectors and variance directions (variable genes with maximum variable percentage) corresponding to the major contributions of the expression profile) (Shen et al., 2006) were plotted in histograms based on transcriptome analysis results for each HCr module in all time-point groups (Supplementary Figure 5). In detail, in the “4,” “8,” “12,” and “48” modules, ***eigengene*** expression levels were highest at 4, 8, 12, and 48 h, respectively, among all the time points after injury examined in this study. In the “20” and “24 modules, ***eigengene*** expression levels were relatively high at 20 and 24 h, respectively (Supplementary Figure 5). Based on ***eigengene*** expression in each selected HCr module, we could use these modules for further analyses.

### Functional Analysis of Gene Expression Profiles in Each HCr Modules Reflecting the Characteristics of Post-SMI Transcriptional Dynamics

After selecting the most relevant meaningful module in each group, we analyzed gene expression profiles to reflect the transcriptional kinetic characteristics of each module. We performed functional analysis to determine the transcriptional dynamics changes during the early post-SMI period.

We focused on biological processes among the GO terms that were enriched in the selected modules and selected the GO terms of interest based on their associated *p*-values (*p* < *0.01*). Due to the unequal numbers of genes in each selected module, the numbers of biologically meaningful GO terms chosen differed among modules (Table 1). The screening process of Kyoto Encyclopedia of Genes and Genomes (KEGG) pathways for each selected module was similar to that described for GO terms. According to the above principles, we focused on several phases of pathological changes in the early period after SMI, Based on this, the GO terms and KEGG pathways screened are shown in Supplementary Figure 6.

**Figure 2:**
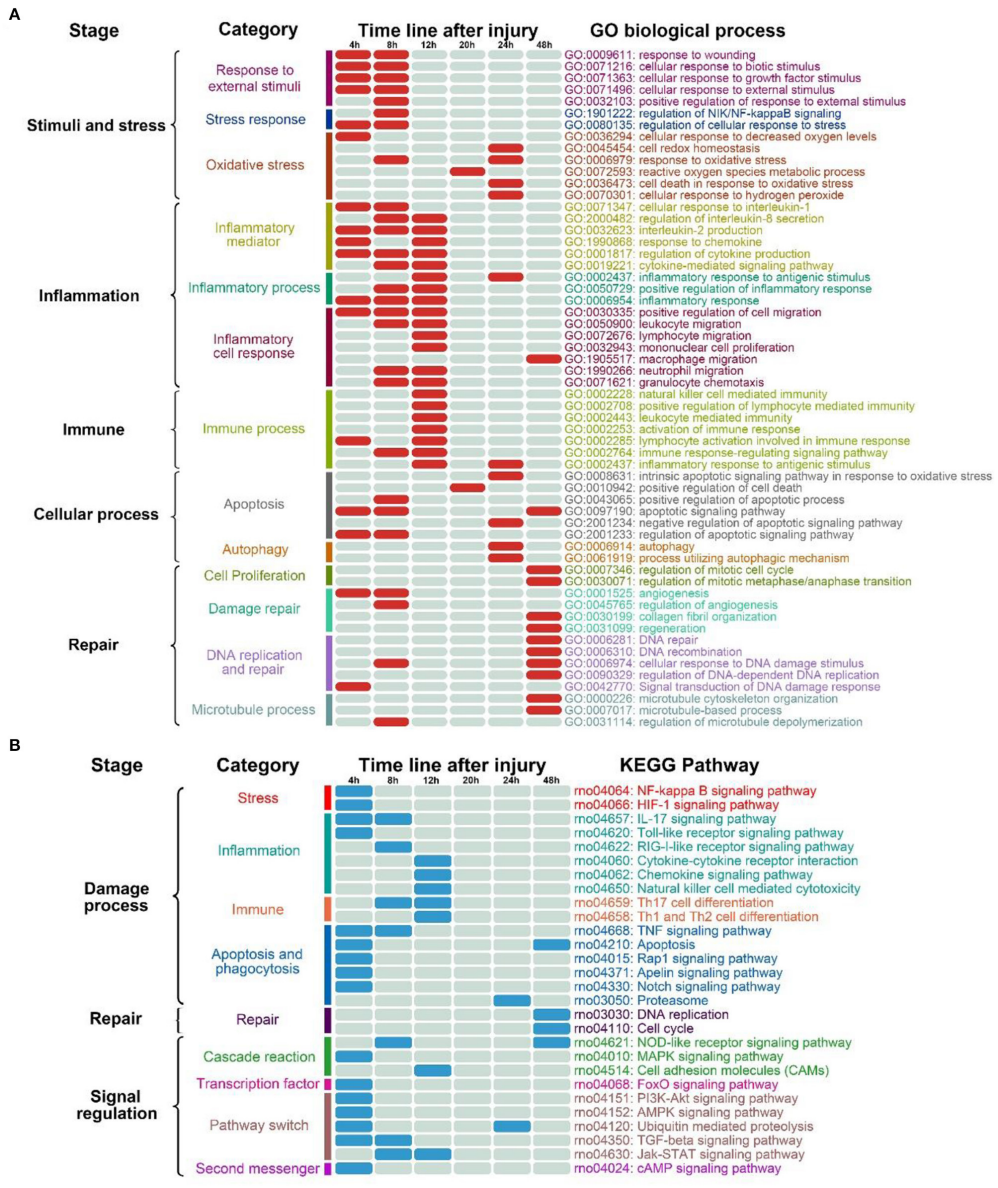
Variation in transcriptional dynamics during the early period after SMI. **(A)** Changes in transcription during post-SMI repair associated with biological processes based on GO analysis. Different colors represent different sub-classes associated with detailed bioprocesses. Further information regarding the GO terms enriched in each sub-class is listed on the right in the same colors. Solid red cells in the table represent the GO terms enriched in the module representing each time-point during the course of injury repair. **(B)** Changes in transcription during post-SMI repair associated with KEGG pathways. Different colors represent different sub-classes associated with detailed pathways. Further information regarding the KEGG pathways enriched in each sub-class is listed on the right in the same colors. Solid blue cells in the table represent the KEGG pathways enriched in the module representing each time-point during the course of injury repair.

As outlined above, the entire course from SMI to repair can be roughly divided into five interconnected biological stages: response to stimuli and stress, inflammation, immune process, cellular process, and repair process (Figure 2A). In the stage associated with stimuli and stress, most GO terms were enriched in the “4” and “8” modules. As shown in Figure 2A, almost all GO terms enriched in the “12” module were associated with inflammation and the immune process. Especially the 48 h module, most of the enriched GO terms were related to the repair process.

During the entire course of the SMI repair process, based on the enrichment of GO terms in all HCr modules, each stage could be further divided into some sub-classes (Figure 2A). In the stage associated with stimuli and stress, the response to external stimuli and the stress began immediately after injury. The oxidative stress responses also began in the early period of the SMI repair process. We divided the inflammation stage into sub-stages associated with the release of inflammatory mediators, inflammatory processes, and the inflammatory cell response.

In particular, among the sub-classes pertaining to inflammatory cell response, the migration of various inflammatory cells (neutrophil, macrophages, lymphocyte, and mononuclear) exhibited a time-varying pattern. From the number of enriched GO terms, we inferred that inflammation predominated from 4 to 12 h after injury. Similarly, in the immune process stage, most immune-related GO terms were enriched in the “12” module. By contrast, autophagy was only enriched in the “24” module (Figure 2A). In addition, the GO terms of DNA repair, recombination, and regulation of DNA-dependent DNA replication were enriched in the “48” module, suggesting the involvement of DNA-related intracellular mechanisms in the course of tissue repair (Supplementary Figure 6A).

Taken together, the findings outlined above demonstrated the temporal variation in GO terms enriched in each HCr module during the entire course of the SMI repair process. The changes in gene expression profiles revealed the basic biological processes and pathological changes occurring during the SMI repair process.

Similar to GO term enrichment analysis, the entire course of the SMI repair process could be roughly divided into stages associated with damage, the repair process, and signal regulation based on KEGG pathway analysis (Figure 2B). We also classified the KEGG pathways into 2–3 sub-classes for each stage of the SMI repair process (Figure 2B). The distribution of KEGG enriched pathways varied in a time-dependent manner. For example, the IL-17 signaling pathway, RIG-I-like receptor signaling pathway, and Toll-like receptor signaling pathway, which are related to the production of inflammatory factors, were enriched in the “4” and “8” modules, whereas pathways related to inflammation and the immune response, such as natural killer cell-mediated cytotoxicity, were enriched in the “12” module (Figure 2B). Pathways related to DNA replication and cell cycle in the repair stage were enriched in the “48” module (Figure 2B). These observations were consistent with the time-dependent variation exhibited by GO terms and the mechanisms underlying the muscle damage-repair process. Taken together, GO and KEGG enrichment analyses for all HCr modules indicated the temporal pathological variation and regulatory processes occurring during the early phase of SMI repair in rats.

In this turn, temporal variation in the active biological processes and pathways after injury were precisely regulated by some aspects of regulation-related KEGG pathways, especially during the initial period of the SMI repair process (i.e., in the “4” module; Figure 2B). The enriched regulatory KEGG pathways included those related to cascade reactions (such as the NOD-like receptor and MAPK signaling pathways); transcription factors (such as the FoxO signaling pathway); pathway switching, including protein phosphorylation regulation and histone modification (such as the Jak-STAT signaling pathway and ubiquitin-mediated proteolysis); and second messenger regulation (such as the cAMP signaling pathway) (Yang et al., 2007; Sharma et al., 2012; Icli et al., 2019).

In summary, throughout the entire SMI repair process, temporal variation in the distribution of KEGG pathways and biological processes (as indicated by GO term enrichment) in each HCr module objectively reflected the transcriptional dynamics as observed in gene expression profiles and the biological significance of the HCr modules (Figure 2).

### Functional Analysis of High-Connectivity Genes in the HCr Modules Illustrates the Features of Post-SMI Transcriptional Dynamics

**Figure 3:**
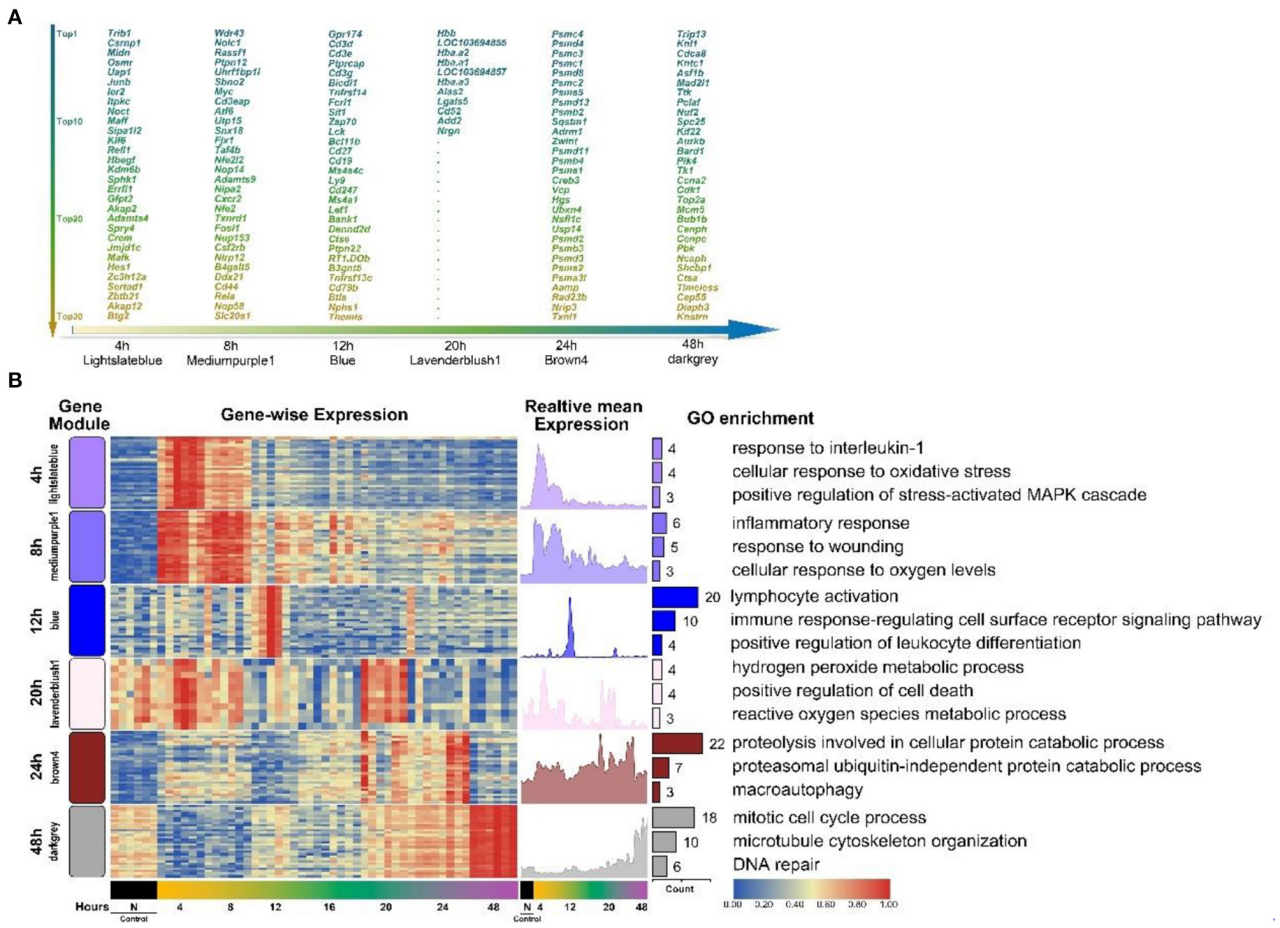
The top high-connectivity hub genes in each HCr module identified using WGCNA analysis. **(A)** The top 30 high-connectivity genes in six time-point-related HCr modules listed by degree of connectivity. Different colors represent the different degrees of connectivity in each module (161 genes in total). Only 11 genes were screened in the 20 h module. **(B)** Clustered heat map of the distribution of the 161 hub genes across time-point-related HCr modules (left, HCr modules are shown in different colors according to WGCNA results). The mean expression in each gene module is shown (right). Functional annotation of the genes in each cluster was performed using GO enrichment analysis. Only the top three enriched processes are shown, sorted by *p*-value.

To further confirm the HCr module selected previously to represent each period, and to study the expression of representative functional genes in each HCr module, we screened the top 30 high-connectivity genes in each HCr module based on the results outlined above. First, we entered all co-expressed genes in each module (Table 1) into the web-based analysis program Cytoscape (Kohl et al., 2011) to screen for the top 30 high-connectivity genes in homologous HCr modules. Due to the differences in numbers of co-expressed genes in each HCr module (Table 1), the screening process yielded a total of 161 genes distributed in the HCr modules (Figure 3A).

We performed detailed enrichment analyses of GO terms representing biological processes and KEGG pathways for these 161 high-connectivity co-expressed genes (Supplementary Figure 7). The bubble diagram of GO terms in Supplementary Figure 7A lists the details of the top three GO terms in each HCr module discussed above (in total, 50 GO terms representing biological processes were screened). As shown in Supplementary Figure 7B, seven KEGG pathways related to the immune response, proteasome, and repair-related process were enriched.

Next, to better show the significance of these genes in each HCr module, a heat map of the top high-connectivity co-expressed genes was produced, which clearly revealed some genes were more highly expressed at specific time points after injury, indicating the relevance of the corresponding HCr module to each time point (Figure 3B). The expression time-series graphs to the right of the heat map showed the relative mean expression of genes in each HCr module, reflecting the significance of these high-connectivity co-expressed genes. Next, GO term enrichment analysis was performed to determine the functional representativeness, of the top high-connectivity co-expressed genes in each HCr module. Among them, we screened the top three representative GO terms in each HCr module to determine the functions of the high-connectivity co-expressed gene cluster and to confirm the transcriptional dynamics in the SMI repair process (Figure 3B). The dominant functions of the co-expressed genes (eigengene in part) in each HCr module matched those expected from the classical biological process of SMI repair illustrated in Figure 2A. Moreover, the HCr modules screened from the results of WGCNA and the high-connectivity co-expressed genes in each module represent the early period of the SMI repair process and reflect the transcriptional dynamics of the gene expression profiles.

### Genes Identified in the Screening Process Represent Time-Series Markers After SMI

Having analyzed the HCr modules identified by WGCNA and expanded on the transcriptional dynamics of the gene expression profiles in all time periods covering the early stages of the SMI repair process, we focused on the time-dependent variation in gene expression. Based on the data analysis workflow (Supplementary Figure 2), we identified time-dependent genes using the maSigPro (microarray Significant Profiles) R package (version 2.0.2) (Nueda et al., 2014).

**Figure 4:**
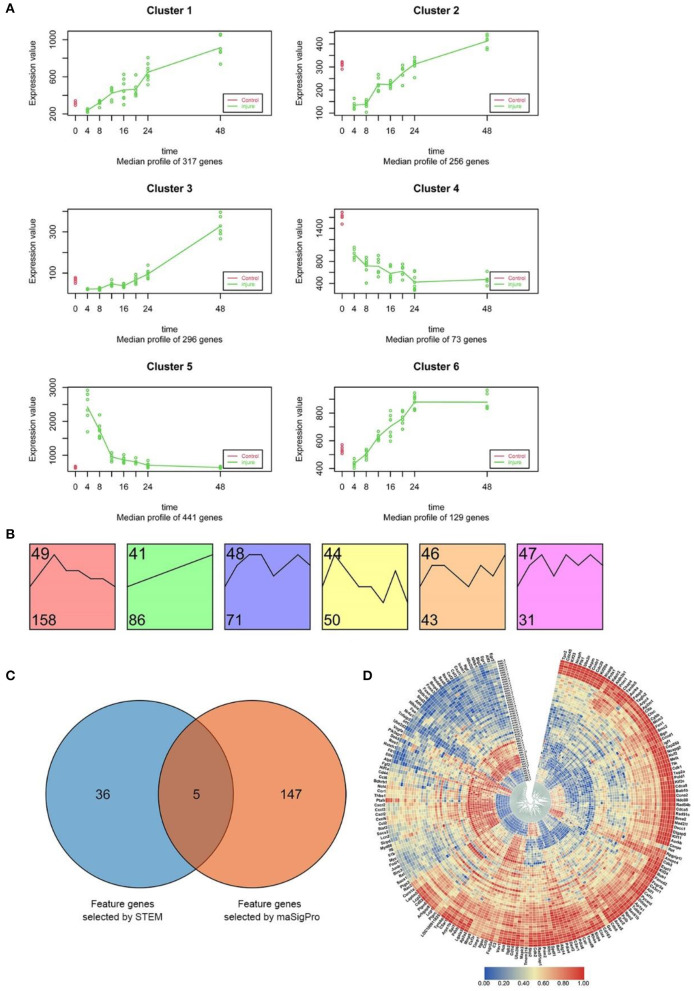
Screening and functional analysis of time-dependent genes using STEM and maSigPro. **(A)** maSigPro results indicating the median gene expression profile for each cluster (green dots and lines represent injury groups; red dots and lines represent control groups). **(B)** STEM analysis of the 540 DEGs (between experimental and control groups) common to the seven experimental groups (*p* < 0.001). The number at bottom left corner indicates the number of genes in each cluster. **(C)** Venn diagram of the genes selected from STEM (41 genes) and maSigPro (152 genes) analyses. We used all genes selected for subsequent analysis (188 genes in total). **(D)** Heat map of the expression of the 188 time-dependent genes selected above in each time period based on hierarchical clustering. Red bars represent high expression levels, blue bars represent low expression levels.

As described above, raw clean data on the experiment-wide gene expression profiles were obtained from 53 samples and 20,662 genes (Supplementary Table 1). The results of maSigPro analysis indicated that during the early period after SMI, 1512 genes exhibited marked temporal changes in expression at the 95% confidence level. Using hierarchical clustering and maSigPro default parameters, these genes exhibiting time-dependent changes in expression were divided into six clusters with different expression profiles during the experiment (Figure 4A and Supplementary Table 3) (Nueda et al., 2014). The raw profiles for each group in each cluster are presented in Supplementary Figure 8A, and the median profile of gene expression and profile differences between the time-dependent experimental and control groups are presented in Figure 4A. In comparison to the control group, the experimental groups exhibited marked downregulation of genes in Cluster 2 and upregulation of genes in Cluster 5. Thereafter, the expression levels of these genes mostly increased in Cluster 2 and decreased in Cluster 5 throughout the whole period after injury, coming close to the levels in the control group. The genes in Cluster 1, Cluster 3, and Cluster 6 were upregulated from 4 to 48 h after injury, whereas the genes in Cluster 4 exhibited the opposite trend (Figure 4A). Due to the different numbers of genes in each cluster (divided according to maSigPro), we chose the high-connectivity genes in the top 10% from the six clusters using a protein–protein interaction (PPI) network visualized with Cytoscape software (version 3.7.2), and a total of 152 genes were selected (Supplementary Figure 8B).

Next, to study the temporal variation in the expression of DEGs at different time points, we performed pattern analysis using STEM software (Ernst and Bar-Joseph, 2006), which has been widely used for in-depth analyses of gene-expression time-series clustering data.

Comparison of the gene expression profiles between the control group and the experimental groups revealed that there were 540 DEGs expressed in common in the seven experimental groups. The distribution of all DEGs in 7 experiment groups (|log_2_FC|≥1) was shown in Supplementary Figure 9A.

These genes are common differential expression gene sets that at each stage of 0–48 h after skeletal muscle injury compared with the control group. According to the GO and KEGG enrichment results of this part of genes (Supplementary Figure 10), the biological functions of the common differential genes at 7 time points after injury enriched mainly included immune performance, inflammation, cell cycle and stress response. From this point of view, according to the literature (Crow et al., 2019), the set of 540 common differential genes can be regarded as systemic global prior differential genes (DE genes).

STEM software was used to identify dynamic gene expression clusters in the common 540 DEGs using default parameters (Supplementary Table 4). The results revealed six expression patterns in the DEGs (*p* < 0.001; Supplementary Figure 9B); however, no similar gene expression trends among these patterns were found (Figure 4B). The analysis also identified 439 common DEGs exhibiting dynamic expression at different time points after injury (Supplementary Table 5). Similar to the results of maSigPro analysis, we then chose high-connectivity genes in the top 10% representing the six expression patterns using a PPI network that was visualized with Cytoscape software, and a total of 41 genes were selected (Supplementary Figure 9C). Furthermore, 41 time-related highly linked genes screened by post-injury common differential genes (global prior differential genes) may play a role in global regulation.

Taken together, the maSigPro analysis based on raw RNA-seq data and STEM analysis based on common DEGs revealed time-dependent marker genes and genes exhibiting dynamic expression during this phase from two perspectives.

### Integrated Functional Analysis of Time-Dependent Marker Genes With Variable Expression Fully Reflects the SMI Repair Process

To comprehensively study the function of the time-dependent marker genes selected as outlined above using an in-depth analysis, we integrated the 152 genes screened by maSigPro and the 41 genes (global prior differential genes) selected by STEM. To obtain marker genes fully reflecting temporal variation during the SMI repair process, we extracted the union set from the total of genes and identified 188 genes for further in-depth analysis (Figure 4C).

This set of 188 genes included marker genes exhibiting time-dependent variation in expression and genes with dynamic expression during the early period after SMI. The circular heat map in Figure 4D indicates that throughout the whole period after SMI, nearly one-third of these genes were downregulated, whereas the remaining two-thirds were upregulated. This observation indicated that these 188 genes exhibited time-dependent variation in expression and could be used as time-dependent marker genes.

We then examined the expression of the 188 time-dependent marker genes using GO term enrichment (biological processes) and KEGG pathway analyses. These 188 genes were associated with 737 GO terms and 48 KEGG pathways. In the GO term enrichment analysis, we selected GO terms based on the *p*-value (*p* < 0.01) and identified 52 GO terms (Supplementary Figure 11A). The selected GO terms indicated that the 188 genes reflected the mechanisms underlying the early stages of the SMI repair process. Then, we identified 42 KEGG pathways, which are listed in Supplementary Figure 11B. The KEGG pathways associated with these 188 genes also reflected the precise regulation of multiple processes after injury, e.g., the TNF signaling pathway, IL-17 signaling pathway, osteoclast differentiation, cell cycle, and chemokine signaling pathway (Supplementary Figure 11B).

In summary, the results suggested that these genes not only reflected time-dependent changes but also included hub genes and core regulatory genes participating in the many complex processes involved in SMI repair.

### Elucidating the Biological Significance of Marker-Gene Expression Changes Using a Combination of Time-Dependent Genes and High-Connectivity Genes

Having illustrated the characteristics of the transcriptional dynamics and time-dependent gene expression variation, in this section, we further illustrate the possible connection between transcriptional dynamics that reflect biological processes and time-dependent marker genes to identify potential biomarkers that will allow us to elucidate the correlations between temporal changes in gene expression and the biological processes that occur in the early period after SMI. In addition, we verified our results based on morphological and immunohistochemical analyses.

**Figure 5:**
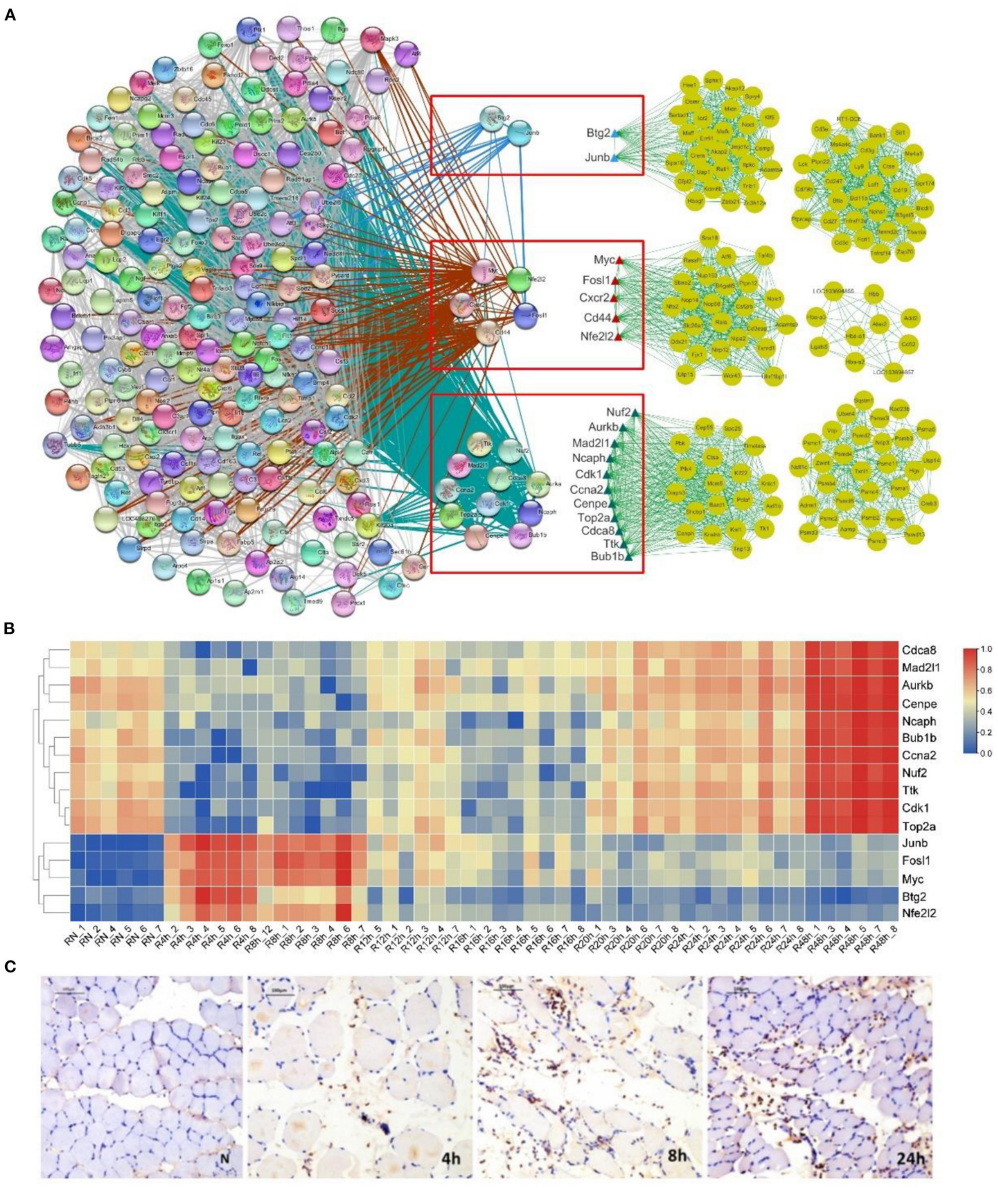
Gene net and heat map of 18 selected gene markers and distribution of neutrophils during the early period after SMI. **(A)** Net of 18 marker genes (red-outlined boxes) selected by the intersecting the 188 time-dependent gene set and 161 hub genes distributed across the six HCr modules identified using WGCNA. The gene network on the left represents the 188 time-dependent genes. The six gene networks on the right represent hub genes selected by WGCNA. The blue, red, and green triangles represent different modules. **(B)** Heat map of 18 marker genes representing their time-dependent expression. **(C)** Immunohistochemical staining for the neutrophil marker MPO. Representative images of transverse sections from three experimental groups and the control group are shown. MPO-positive neutrophils are indicated by brown particles. Scale bar: 100 μm.

First, we identified the genes in common between the 161 high-connectivity co-expressed genes (eigengenes) in the HCr modules and the 188 time-dependent marker genes. The results indicated that there were 18 genes common to the two gene sets (Figure 5A). Next, we analyzed the positions of these 18 genes in a network in which the set of 161 high-connectivity co-expressed genes and the set of 188 time-dependent marker genes were combined, as well as the functions of the 18 genes during the early period after SMI. In Figure 5A, the 18 genes are listed in a red-outlined box in the middle of the two gene networks. Genes in these two networks are connected by lines of different thickness. The functions of the 18 genes also corresponded to the main biological processes discussed above, and they may be hub genes in these complex processes after injury (Figure 5A and Table 2). In addition, we produced a heat map of the 18 genes to visualize changes in their expression levels during the early period after SMI. The expression levels of the bottom one thirds of genes from the “4” and “8” modules were initially upregulated, and then gradually downregulated. By contrast, the expression levels of the top two thirds of genes from the “48” module exhibited the opposite trends (Figure 5B).

**Table 2:** Detailed information and main functions of 18 marker genes.

**Gene Name**	**HCr module**	**Marker**	**Gene functions**
*Btg2*	4 (lightslateblue)	Blue triangle (2 genes)	Participation in cell differentiation and apoptosis; transcriptional regulation (Tirone, 2001)
*Junb*	4 (lightslateblue)	Blue triangle (2 genes)	Involved in angiogenesis; transcriptional regulation (Zou et al., 2018)
*Myc*	8 (mediumpurple)	Brown triangle (5 genes)	Promotion of skin wound healing (Wang et al., 2020)
*Fosl1*	8 (mediumpurple)	Brown triangle (5 genes)	Angiogenesis and cell proliferation regulation (Galvagni et al., 2013)
*Cxcr2*	8 (mediumpurple	Brown triangle (5 genes)	Mediation of the migration of neutrophils to inflammation sites (Donahue and Hines, 2009)
*CD44*	8 (mediumpurple)	Brown triangle (5 genes)	Mediation of leukocyte adhesion and migration; macrophage subpopulation regulation (Krolikoski et al., 2019)
*Nfe2l2*	8 (mediumpurple)	Brown triangle (5 genes)	Promotion and regulation of the oxidative stress response in the inflammation process (Arefin et al., 2020)
*Top2a*	48 (dark-gray)	Green triangle (11 genes)	Participation in DNA repair (Swan et al., 2020)
*Ttk, Cdca8*	48 (dark-gray)	Green triangle (11 genes)	Regulation of cell proliferation (Li et al., 2020)
*Nuf2, Aurkb, Mad2l1*	48 (dark-gray)	Green triangle (11 genes)	Cell cycle regulation; cell mitosis regulation (Hegyi and Méhes, 2012; Zhang et al., 2019a)

These observations indicated that the transcriptional dynamics of the co-expressed genes in the HCr modules were closely related to the time-dependent marker genes through these 18 genes. In addition, based on the integration of transcriptional dynamics and the time-dependent marker genes, some of the hub genes may reflect temporal gene-expression changes associated with biological processes occurring in the early stage following SMI.

Next, to intend confirm the process of inflammation during the early period after SMI, we labeled neutrophils using an anti-MPO primary antibody. The results of immune-histochemical analysis suggested that, in comparison with the normal control group, with the development of inflammation, the degree of neutrophil infiltration and the neutrophil distribution varied in a characteristic manner during the early period (0–24 h) after SMI (Figure 5C).

Taken together, the results of the histo-morphological and immune-histochemical analyses reflected the characteristics of the SMI repair process.

## Discussion

SMI repair is a continuous and dynamic biological process (Chellini et al., 2019). Throughout the whole process, especially in the early stages after injury, changes in tissue morphology, cell proliferation, gene expression, and pathway regulation are rapid and complex (Sass et al., 2017; Liu et al., 2020). There have been a number of studies regarding the connections between a variety of complex biological processes and specific time periods after injury. In addition, systematic approaches for analysis during this dynamic biological process have been developed. Gene expression in biological systems is finely regulated. On the whole, altering the genes and regulation factors that maintain the state of the system causes changes in some of the genes in it. On the other hand, after the system is disturbed, the control genes in the initial state are consumed and start to change, thus causing the change of gene expression in the system (Efroni et al., 2008).

However, there is still a lack of genes or biomarkers that can be used to clearly distinguish the various stages after injury and for monitoring the continuous and dynamic biological processes. Using next-generation sequencing technology and time-series algorithms, many studies have conducted systematic and transcriptional dynamics analyses to understand the time-dependent progressive development of physiological processes and time-dependent gene expression in biological processes, such as those involved in transcriptional variation in skin wound healing (Theocharidis et al., 2020), changes in transcription profiles during healing after fractures (Coates et al., 2019), changes in transcriptional dynamics during oral and liver tumor progression (Jee et al., 2019; Kang et al., 2019), and changes in the transcriptome during organ and tissue development (Zhu et al., 2018). In this study based on next-generation sequencing, we used WGCNA co-expression module analysis, STEM and maSigPro time-related algorithms, and PPI network analysis to clarify the characteristics of multi-level biological processes and transcriptional dynamics after SMI.

From the perspective of early transcriptional dynamics after injury, in the “4” and “8” modules, most enriched GO terms were associated with the response to injury stimulation and stress (Filippin et al., 2009), inflammatory mediator release, and inflammatory responses. Furthermore, inflammatory response to antigenic stimulus and macrophage migration were enriched in the 24 and 48 h HCr modules, respectively, suggesting that inflammation occurs throughout almost the entire SMI repair process. However, the apoptotic signaling pathway, positive regulation of the apoptotic process, and the intrinsic apoptotic signaling pathway in response to oxidative stress became active in the early post-injury period and may persisted until 48 h or even later after injury. In addition, KEGG pathways related to a single regulatory process were mainly enriched in the “4,” “8,” and “24” modules. This indicated that the bioprocesses were regulated throughout the whole period after injury (Figure 2B). In the early period after injury, the tissue repair process was mainly focused on vascular regeneration. While, the process of tissue repair occurred mainly in the period from 48 h or later during the SMI repair process. Fibrous repair, tissue regeneration, and cell proliferation were the main repair pathways (Figure 2A).

In this study, we refined the transcriptional characteristics of the early stage after SMI. GO and KEGG analyses of the HCr modules showed that this dynamic stage involves multiple continuously changing biological processes after SMI, including connections between related regulatory factors, transcription factors, protein modification changes and pathway regulation. The results of histomorphological and immune-histochemical analyses (Figures 1A, 5C) also revealed morphological changes in damaged muscle tissue and the infiltration and migration of inflammatory cells (neutrophils) around the tissue at 0–48 h after SMI, reflecting the temporal variation inherent in the SMI repair process. Above all, these findings contribute to further research on wound age estimation from the perspectives of pathways, protein modification, pathway regulation, and cell migration.

Yang et al.'s study found that *Btg2* may be a target gene for miR-222-3p, and during myogenesis, the expression of *Btg2* in C2C12 myoblasts regulates cell proliferation and differentiation (Yang et al., 2019). In this study, we found that *Btg2* distributed in the 4 h module after skeletal muscle injury. This suggested that *Btg2* may promote the differentiation and maturation of myoblasts in the early stage after skeletal muscle injury. For this turn it might play a role in the regeneration and repair for injured skeletal muscle. In addition, Junb gene plays an important role in functional recovery of damaged skeletal muscle, promoting regeneration of damaged skeletal muscle cells, and regulating remodeling and functional differentiation of skeletal muscle cells during skeletal muscle development (Li and Luo, 2019). In this study, similar to *Btg2, Junb* might play a major role in the regulation and maintenance of skeletal muscle cell size and remodeling after skeletal muscle injury. Furthermore, the c-Myc related genes distributed in the 8 h module in this study, can inhibit the differentiation of myoblasts and promote the proliferation of myoblasts and muscle fiber hypertrophy (Luo et al., 2019). Studies showed that MyoD1 may regulate the transition from differentiation to proliferation of skeletal muscle cells through *Myc* protein (Kohsaka et al., 2014). For this case, *Myc* and myosatellite cell-related protein MyoD may jointly promote the proliferation of damaged skeletal muscle and play a role in skeletal muscle regeneration. In the future study of the repair process initiated at the early period after skeletal muscle injury, the relationship between *Myc* and MyoD deserves further attention.

From the perspective of systems biology and gene expression dynamics, in the early period of skeletal muscle injury-repair process, when the damage occurs, the system was disturbed by exogenous disturbance, which destroys the initial critical state of the gene and transforms from near critical state to supercritical state. From this perspective, the genetic reprogramming occurs to drive changes in system state and biological phenotypes (Tsuchiya et al., 2015). Gene expression state in the same tissue is relatively stable as a whole and is maintained by subcritical genes and genomic attractors (Tsuchiya et al., 2016). In this case, WGCNA was used to divided gene expression matrix after SMI into time-dependent modules, and the gene expression in each module had two forms of transformation and maintenance during this process. While the same tissue (skeletal muscle) gene under conditional perturbation (damage), over time, leads to the genome-wide attractor deviation, and the random coherence occurs (Tsuchiya et al., 2015). In this view, the changes in gene expression and system state after skeletal muscle injury may depend on the occurrence of such random coherence.

Our analysis indicated that many biological processes were involved and the active processes changed frequently within 0–12 h after SMI. However, in comparison with other time periods, no obvious gene-expression module was distinguished at 12–16 h after injury. This time period may represent a transition stage from stress inflammatory response to tissue remodeling Moreover, as can be clearly seen in Figures 2A,B, there were fewer enriched GO terms and no enriched KEGG pathways from 16 to 20 h after injury. Therefore, we believe that there may be a plateau in gene expression and biological processes within a certain period after SMI.

In this study, among the injured skeletal muscle tissue, the eigengenes and hub genes within the HCr modules might be in a supercritical state during the state transition process, and other regulatory genes, including some housekeeping genes and tissue stability genes within the module were involved in the maintenance and drive of the system state (Tsuchiya et al., 2016). From this perspective, we could explain the possible mechanism of gene expression changes in the phase of 16–20 h after skeletal muscle injury in this study. The results showed that the significance modules could not be divided through the threshold value at 16 h after injury, so there were not enough module eigengene drive system states variation during this period.

The transition of the whole system state depends not only on the local regulation of genes, but also on the global regulation. The local regulation of eigengenes in module or hub genes within the module drove the system state transformation. Meanwhile, from a global perspective, the expression of low and moderately mutated DE genes in the whole system played an important role (Tsuchiya et al., 2014). Based on this, in our study, we analyzed and described the changes of gene expression in the early stage of skeletal muscle injury and the key genes that driving the transition of the state from the perspectives of the changes in the module eigengenes from the gene expression profile spread over time HCr modules after skeletal muscle injury and the regulation of time-dependent differential genes and global common DE genes. Meanwhile, we have broadened the robustness of the screening threshold for DE genes selection.

In addition, the interaction between genes (gene network) will change during the occurrence of diseases or specific biological processes driven (Censi et al., 2011). Hub genes which were selected from multiple time HCr modules and time-related gene clusters represented the gene interrelationship after injury in a specific state. In addition, due to the changes of intergene relationships after skeletal muscle injury, we have reason to believe that the changes of temporal intergene relationships and environmental factors lead to the variation of gene criticality in the system, which may be another cause of the changes of gene expression profile and phenotype after skeletal muscle injury that deserves further study.

In summary, changes in gene expression profile, complex phenotypes, and time-related genes after skeletal muscle injury are correlated with changes in local module characteristic gene states, global gene expression regulation, and gene interaction networks (changes in hub genes) in skeletal muscle tissue biosystem. Interestingly, during 24–48 h, especially at 48 h after injury, more intramedular eigengenes were screened out (Supplementary Figure 5), which may be driving the key transition of skeletal muscle tissue from injury to repair process. It can be inferred that the gene expression level changed greatly in this period after injury, and then the key transformation of the driving system might occur at this stage. This key transformation is related to the significant changes in the gene interaction network (the number of hub genes increased) at this stage and the possible construction of some new gene regulatory networks. Furthermore, it seems to provide clues for further research on the initiation of skeletal muscle injury repair process during the early stage after skeletal muscle injury.

For another part, during the damage-repair process post-SMI, the dynamic changes based on transcriptional kinetics and data on the time-specific and time-related gene screening obtained via the application of time-related algorithms have high reference value, especially for forensic research on wound age estimation. Additionally, we have information on methods for the examination of initial tissue morphology (Yagi et al., 2016), the quantitative analysis of labeled proteins (Zhao et al., 2009), combined PCR analysis of multiple indicators (Du et al., 2013), and more recently, combining multiple indicators and multiple statistical analysis approaches (Du et al., 2020). In this study, instead of a theoretical algorithm, we developed a method for selecting time-dependent indexes using omics data. Combined with information on time-related gene modules, we screened for more accurate and objective time-dependent indicators that reflect the damage-repair process. This provides new possibilities for shortening the time inference window in the early period after injury.

In addition, regarding the 18 network node genes (Figure 5A) that were finally identified in this study, many groups have shown that they play a significant role in the regulation and linkage of related pathways in trauma, skin wound healing, inflammatory processes and inflammation-related diseases, tumor progression, and other inflammation-related biological repair processes (Han et al., 2019; Kumar et al., 2020; Wang et al., 2020) (Table 2). Studies showed that among *Fos* proteins, including *Fosl1*, significantly bind to the promoter regions of multiple genes and differentially regulate the expression level of related genes during the processes of tissue and cell damage, repair and differentiation (Reddy and Mossman, 2002). In addition, *Fosl1* plays an important role in the regulation of cell proliferation, differentiation, apoptosis, movement, invasion and metastasis, and it can increase cell adhesion and inhibit cell migration after tissue injury (Galvagni et al., 2013). Not only *Fosl1*, but *CXCR2* also plays a key role in the regulation of neutrophil movement and distribution (Zuñiga-Traslaviña et al., 2017). In studies on a variety of inflammatory diseases and tissue damage showed that *CXCR2* might be used as a potential target for early anti-inflammatory intervention (Zhu et al., 2020). According to the results of immunohistochemistry in this study, the migration and distribution of neutrophils after skeletal muscle injury generally showed a certain regularity in morphology aspect. At the same time, combined with *CXCR2*, the regulation hub of the net of the module genes and showed the time-dependent change pattern, we can further pay attention to the regulation effects of related genes and markers on neutrophil movement and distribution during the early period after skeletal muscle injury. Meanwhile, researches showed that *CD44* interacts with hyaluronic acid (HA) to regulate reprogramming of pro-inflammatory macrophages (M1) to anti-inflammatory macrophages (M2). The transformation of macrophage polarization from inflammatory (M1) to anti-inflammatory (M2) phenotype has significant significance for the regeneration of damaged tissues, the treatment of inflammatory diseases and the remission of autoimmune diseases (Shahbazi et al., 2018). Above all, from the perspective of the regulation of inflammatory cell migration and phenotypic transformation, further exploration of the transformation regulation of pro-inflammatory and anti-inflammatory response during the early state after skeletal muscle injury is expected to be a new direction for early intervention of skeletal muscle injury.

These genes may be of value not only in studying gene expression and transcriptional regulation mechanisms in damage-repair processes but also as references in research regarding wound age estimation and in reflecting the dynamic continuous processes of damage repair.

Moreover, translational medicine research is becoming an area of major importance in the post-genomic era (Hegyi et al., 2020). From the perspective of homologous gene expression and conservation through evolution, the study of diseases or physiological processes based on time-series changes in model organisms lays a foundation for cross-species inference or translational research among related species (Parikh et al., 2010; Hardison, 2016; Zhu et al., 2018). Based on detailed studies of gene expression and transcription kinetics in biological processes, research at the level of gene-expression changes after injury has significant reference value for research on translational medicine and for the further exploration of cross-species translation based on the same or similar biological processes among related species (Czarnewski et al., 2019; Hansen et al., 2020).

In this turn, for studies among different species are essentially in different systems, based on changes in system state and gene expression in similar biological processes under the same disturbance. From the above analysis, in the future, we should not only analyze the phenotypes and gene changes of the state after the disturbance, but also further explore the similarity of the changes of the state after skeletal muscle injury among species from the perspective of local and global regulation and the variation of interaction between genes. In this way, we can further explore the wound age estimation and the repair of skeletal muscle injury among different species from the perspective of key genes, global regulation and the changes of interaction between genes.

Further, we will use multi-scale statistical models to quantitatively integrate the changes in local gene associations and global gene networks in order to find more valuable key genes that influence system dynamics and driver gene expression and phenotypic changes in the early period after SMI. Based on multiple time points after injury of common DE genes as an important role of global prior DE genes (Harris et al., 2016). In the follow-up study, to this part of the genes, we will use to distinguish between damage samples with different time period after injury for wound age estimation. Further, to explore and validate the key role of global prior DE genes in wound age estimation and to drive the stages transition after skeletal muscle injury, especially the damage to repair phase.

## Conclusions

In this study, we applied next-generation sequencing technology in combination with bioinformatics analysis and time-correlation algorithms to explore the transcriptional dynamics in the 0–48 h period after SMI. Moreover, we characterized the dynamic changes in biological processes with specific time periods and detailed the transcript features and complex biological processes in the early stages after SMI. Using time-dependent analysis software and algorithms, combined with WGCNA to identify time-correlated gene expression modules, 18 biologically significant time-correlated change indicators were identified in the screening process. This is an important basis and reference for follow-up studies on the mechanisms operating in the early period of the SMI repair process, clinical diagnosis and treatment for SMI, forensic wound age estimation, and for the future translational medical research.

## Materials and Methods

### Experimental Animals and Models

Male 6–7-week-old Sprague Dawley rats were obtained from the Experimental Animal Center of Shanxi Medical University (Taiyuan, Shanxi, China) and housed in environmentally enriched ventilated cages under specific pathogen-free conditions at the Provincial Key Laboratory of Forensic Medicine, Shanxi Medical University (Jinzhong, Shanxi, China) under a 12h light/dark cycle with *ad libitum* access to water and food [RM1 (P); Regular Diet Services]. Sixty-four male Sprague Dawley rats were divided randomly into eight groups (seven experimental groups and one control group, *n* = 8 rats/group) once their body weight reached 240 g (±20 g) (Figure 1B). The animals in the experimental groups were anesthetized with 10% chloral hydrate (2.5 ml/kg body weight, intraperitoneal injection) and the hair on their right posterior limb was removed using a depilatory agent. A 500 g weight was dropped from a height of 50 cm onto the right hind limb with a free fall motion. The rats in the experimental groups were sacrificed at 4, 8, 12, 16, 20, 24, or 48 h after injury using a lethal dose of 10% chloral hydrate (4 ml/kg body weight, intraperitoneal injection). The animals in the control group were directly sacrificed using the same method as described above in the middle of the 0–48 h post-injury period defined in this study. A muscle sample of ~200 mg was dissected from the wound site of each rat and divided into two parts: (1) samples from the central area of damage (~100 mg) were snap-frozen with liquid nitrogen and cut into small squares for histological analysis after fixation with formalin solution; and (2) the remaining samples (~100 mg) were frozen immediately in liquid nitrogen and saved at −80°C until RNA-seq (50 mg) analysis after the fascia was removed.

### RNA Extraction, Quality Control, and Next-Generation Sequencing

Frozen muscle samples (~50 mg each) were pulverized in liquid nitrogen with an RNase-free mortar and pestle, and then dissolved using RNAiso Plus 9108 (Takara Bio) in accordance with the manufacturer's instructions. The concentration (ng/mL) and purity of the freshly extracted total RNA were measured using a microplate reader (Infinite M200 Pro; Tecan). The quality of the extracted total RNA was determined using spectrophotometry (NanoDrop 2000; Thermo Fisher Scientific). The concentration of total RNA was determined using fluorometry (Qubit 3.0; Life Technologies) and the integrity of total RNA was determined with an Agilent 2100 Nano 6000 Assay kit on an Agilent 2100 Bioanalyzer system. Only RNA samples with OD_260_/OD_280_ ratios within the range 1.8–2.2 and an RNA integrity number > 7.0 were used in the following experiments. Samples not meeting these criteria were excluded from the analysis (*n* = 4).

Sixty samples were submitted to Illumina for library preparation using a NEBNext Ultra RNA Library Prep Kit (poly-A selection; NEB) and fluorometer (Qubit 3.0; Life Technologies) to build and preliminarily quantify the library. The library was then quantified accurately (IQ SYBR GRN Kit; Bio-Rad) and the insert size of the library was determined (Agilent 2100 Bioanalyzer). The library of 60 samples was then subjected to pair-end 150-bp sequencing, aiming for coverage of 40–60 M reads, using a HiSeq PE Cluster Kit v4-cBot-HS on the HiSeq-2500 platform (Illumina). The off-machine data were converted into raw sequence reads after base recognition using bcl2fastq2 software (Love et al., 2014), and the results were stored in the FASTQ file format. The read quality was then inspected using FastQC and MultiQC (Zhou et al., 2018), and trimmed with Trimmomatic (version 3) (Bolger et al., 2014). These procedures yielded clean data for bioinformatics analysis.

### Bioinformatics Analysis of Transcriptome Data

Further bioinformatics data analysis was performed using the high-throughput data analysis software Chipster (version 3.16) (Kallio et al., 2011). The clean paired-end RNA-seq data obtained with Trimmomatic were aligned to the rn6 rat genome (HISAT2 version 2.1.0) (Kim et al., 2015) and annotated based on the Rattus_norvegicus.Rnor_6.0.95^1^ file. In this study, the alignment files contained paired-end data. The mapped and aligned reads were quantified to determine gene-level counts using uniquely aligned unambiguous reads in HTSeq (version 0.6.1) (Anders et al., 2015) with the default settings and reverse strandedness. Raw counts were processed using the R bioconductor package DESeq2 (version 1.12.4 in R Studio version 3.6.3) (Love et al., 2014) and normalized using the DESeq method to remove library-specific artifacts. Among the 53 samples, total absolute read counts of <5 genes were considered to have low expression and were filtered out. DEGs between the experimental and control groups were calculated using the Wald test in DESeq2. Genes with log_2_-fold change (FC; Injured/Control) > 1 or < −1 and adjusted *p* < 0.05 corrected for multiple testing using the Benjamini–Hochberg method (Benjamini and Hochberg, 1995) were considered significant and subjected to further downstream analysis.

### Module Identification Using WGCNA

WGCNA was performed to identify the gene co-expression modules at each post-injury time point using the WGCNA package in R Studio (version 3.6.3) (Langfelder and Horvath, 2008). For subsequent analysis to generate modules, the same parameters were used to construct all modules for each post-injury time point in independent analyses. A signed weighted correlation matrix containing pairwise Pearson correlation coefficients between all genes across all samples was computed using a soft threshold of β = 22 to attain a scale-free topology (Bogenpohl et al., 2019). Using this adjacency matrix, the topological overlap measure, which measures the network interconnectedness (Yip and Horvath, 2007), was calculated and used as an input to group highly correlated genes together using average linkage hierarchical clustering. The WGCNA dynamic hybrid tree-cut algorithm was used to detect network modules of co-expressed genes. After we have selected the aforementioned HCr modules (Jin et al., 2019), functional enrichment analysis of genes in the HCr modules was performed to detect enriched KEGG pathways and GO terms representing biological processes, and statistically significant clusters were identified using the Metascape^2^ database (Zhou et al., 2019).

In gene co-expression networks, high-connectivity genes, referred to as hub genes (high degrees of connectivity), are critical for the maintenance of overall network stability. In WGCNA, intra-module connectivity and correlations with module eigengenes were used to select hub genes without any statistical criteria (Bi et al., 2015). STRING was used to construct a PPI network, with closely related genes located closer together. In the present study, the degrees of hub genes in each HCr module were calculated and visualized using Cytoscape version 3.7.2^3^. The top 30 genes according to degree of connectivity were screened as high-connectivity co-expressed genes in each HCr module.

### Time-Dependent Marker Gene Analysis Using maSigPro

Time-dependent marker gene analysis was performed using normalized log_2_ gene expression values after DESeq analysis using the Next maSigPro R package (version 1.6.0) (Nueda et al., 2014). Statistical analysis of time-series data identified genes that exhibited changes in their expression over time and/or followed a specific expression pattern. Briefly, the p vector function was used to compute a regression fit for each gene in both control and separate experimental groups. Temporally DEGs were detected using the generalized linear model setting with *p* ≤ 0.05 after false discovery rate correction. The final selection of temporally DEGs was performed by filtering the results of the second regression model using the get siggenes function, with the *R*^2^ parameter set to 0.7 and the vars parameter set to groups (Grilli et al., 2018). Finally, significant genes were grouped into k = 6 groups (set value).

As mentioned above, STRING was used to construct a PPI network to select the top 10% of genes in each cluster according to degree of connectivity, and the results were visualized using Cytoscape.

### Time-Dependent Marker Gene Analysis of DEGs Using STEM

STEM (Short Time Series Expression Miner, version 1.3.12) (Ernst and Bar-Joseph, 2006) was used to identify the dynamic gene-expression clusters among the DEGs common to all seven experimental groups, and significantly enriched gene families with similar expression patterns were clustered according to the default parameters. Similar to how genes were clustered, we used STRING to construct a PPI network to select the top 10% of genes in each cluster according to degree of connectivity, and the results were visualized using Cytoscape. Functional GO term and KEGG pathway enrichment analyses of the significant clusters were performed using the Metascape database (see above).

### Histological Analyses (H&E Staining and Immunohistochemistry)

The injured limbs from the experimental groups (*n* = 56) and control group (*n* = 8) were harvested and placed in 10% neutral buffered formalin for 24 h. The muscle tissue was rinsed and flushed with phosphate-buffered saline, gently squeezed to remove non-adherent bacteria, and embedded in paraffin. Then, longitudinal sections 5 μm thick were cut for histological observations and immune-histochemical processing. Histological sections for each time point were stained with H&E to observe the progression from damage to repair in the early period following SMI.

A primary antibody against MPO (1:500 dilution, ab9535; Abcam) was used with a Metal Enhanced DAB Chromogenic Kit (AR1026; Boster) to stain infiltrating neutrophils. A secondary anti-mouse/rabbit horseradish peroxidase immune-globin G antibody (SA1020; Boster) was used to identify sites of inflammation. Sections were counterstained with hematoxylin.

Following staining, the slides were imaged using a Tissue Fax Plus 2000 slide scanner (Tissuen Gnostics) at a magnification of 40× (H&E staining) or 20× (immune-histochemical staining).

## Data Availability Statement

The RNA-seq raw data upload information link, repositories and accession number in the article are listed as follows: Information link: https://www.ncbi.nlm.nih.gov/geo/query/acc.cgi?acc=GSE171243, Repository: GEO Accession number: GSE171243.

## Ethics Statement

The animal study was reviewed and approved by Animal Ethics Committees of Shanxi Medical University [reference number 2016LL151].

## Author Contributions

JS and YW conceived and supervised the project. KR, JS, and QD designed the research. LiangW, NL, LD, LianglW, and YT performed the animal and RNA experiments. LianglW and GA performed data and bioinformatics analyses. JC, LiangW, and KR performed the HE and IHC staining. LianglW and QJ performed the figures on this paper. JS, YW, QD, KR, and NL interpreted the results. KR, JC, and JS wrote the paper. All authors contributed to the article and approved the submitted version.

## Conflict of Interest

The authors declare that the research was conducted in the absence of any commercial or financial relationships that could be construed as a potential conflict of interest.

## Footnotes

^1^http://hgdownload.soe.ucsc.edu/downloads.html#rat

^2^http://metascape.org/gp/index.html#/main/step1

^3^http://apps.cytoscape.org/apps/iRegulon
