# Supplementary material for: Investigating Transcriptional Dynamics Changes and Time-Dependent Marker Gene Expression in the Early Period After Skeletal Muscle Injury in Rats
Source: Front Genet. 2021 Jun 17;12:650874. doi: 10.3389/fgene.2021.650874 (PMC8248501; doi:10.3389/fgene.2021.650874)
Supplement: Supplementary file 1 [file Presentation_1.pdf]

Supplementary Materials for

Investigating transcriptional dynamics changes and time-dependent marker gene expression in the early period after skeletal muscle injury in rats

This PDF file includes:

Figs. S1 to S11

Captions for Tables S1 to S5

Other Supplementary Materials for this manuscript includes the following:

Table S1 to S5 (Excel)

Supplementary Fig.1

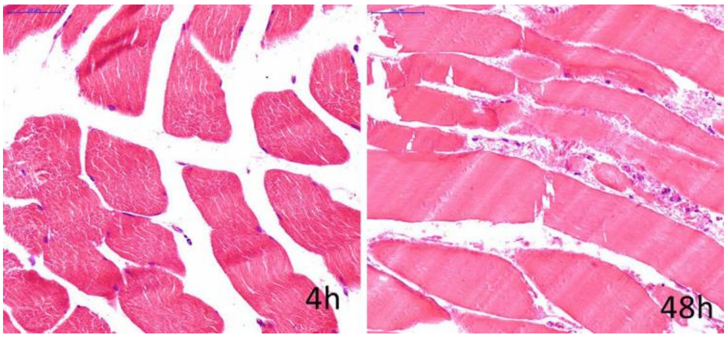

Fig.S1 Histomorphology evaluation of the longitudinal section in skeletal muscle after injury. The HE stain showed the classic process of the injury (4h post injury) to repair (48h post injury) in longitudinal section of the muscle fibers. Scale bar for 50μm

Supplementary Fig.2

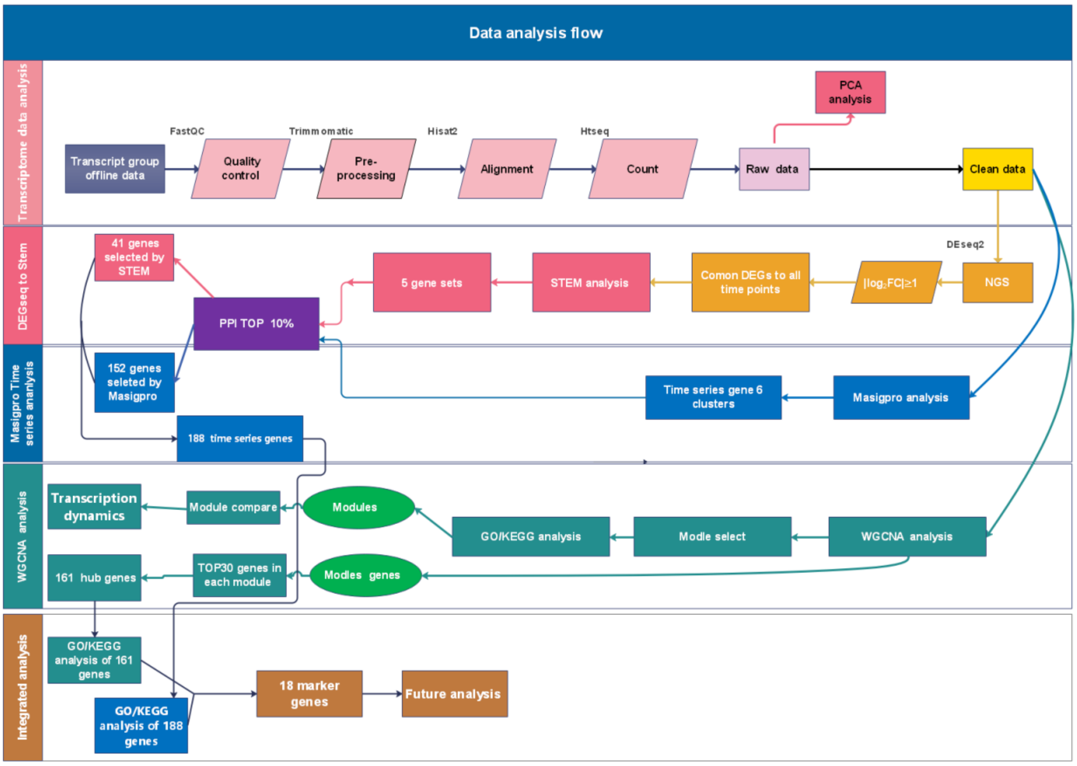

Fig. S2 Data analysis flow chart. The processes and research design for analyzing transcriptional dynamics characteristics and time-dependent marker gene screening after SMI in rats are shown. Each lane represents one type of data analysis, and the lanes are connected by arrows of different colors. PPI, protein–protein interaction; NGS, next-generation sequencing; KEGG, Kyoto Encyclopedia of Genes and Genomes.

Supplementary Fig.3

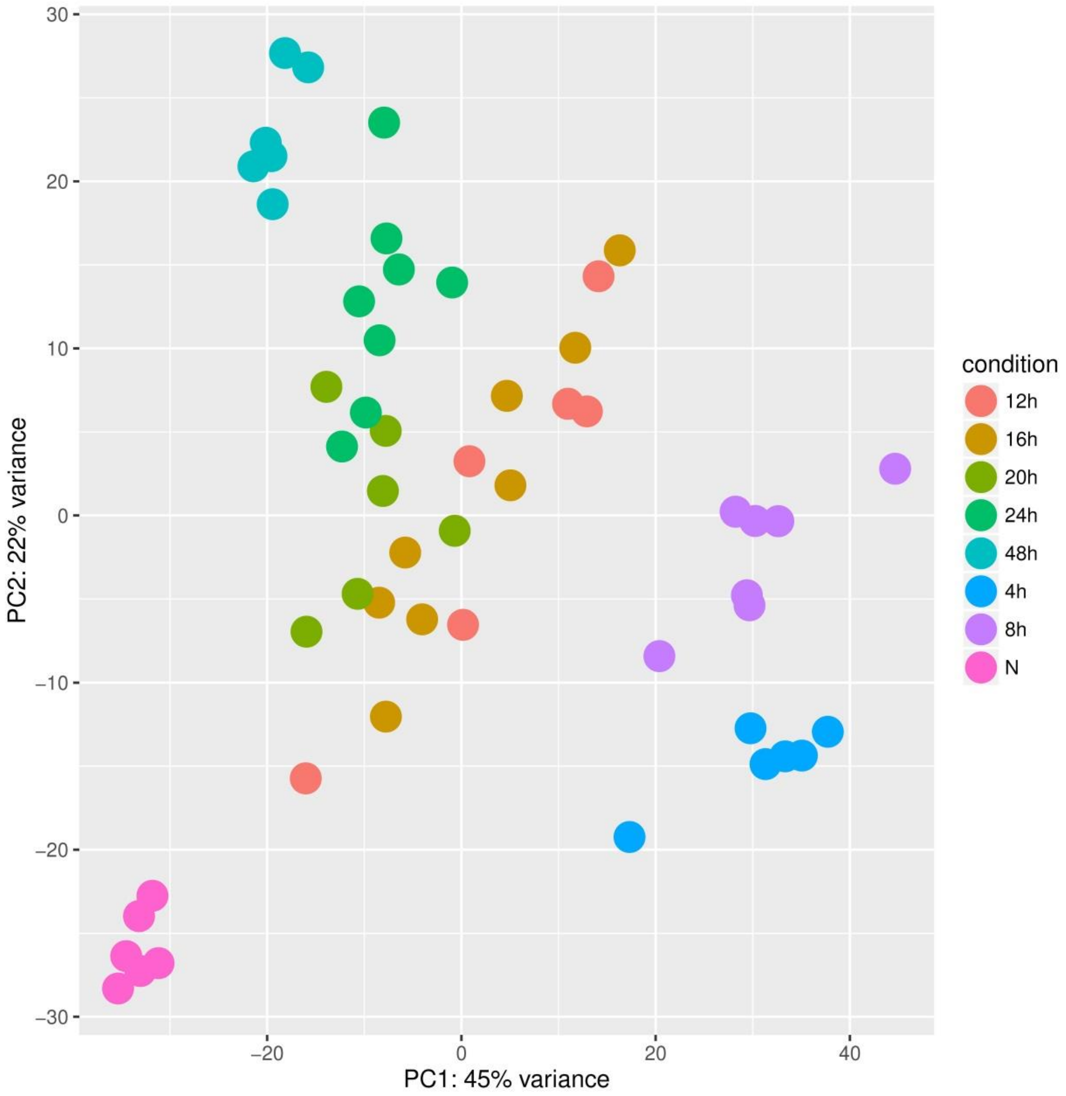

Fig.S3 Principal component analysis (PCA) shows the clustering of samples in each group The PCA shows the clustering trend of samples divided in each experimental and control group based on the raw genes expression data in this study. Different color circles clustered samples in different groups. The dots with different colors represent the samples clustered in one group.

Supplementary Fig.4

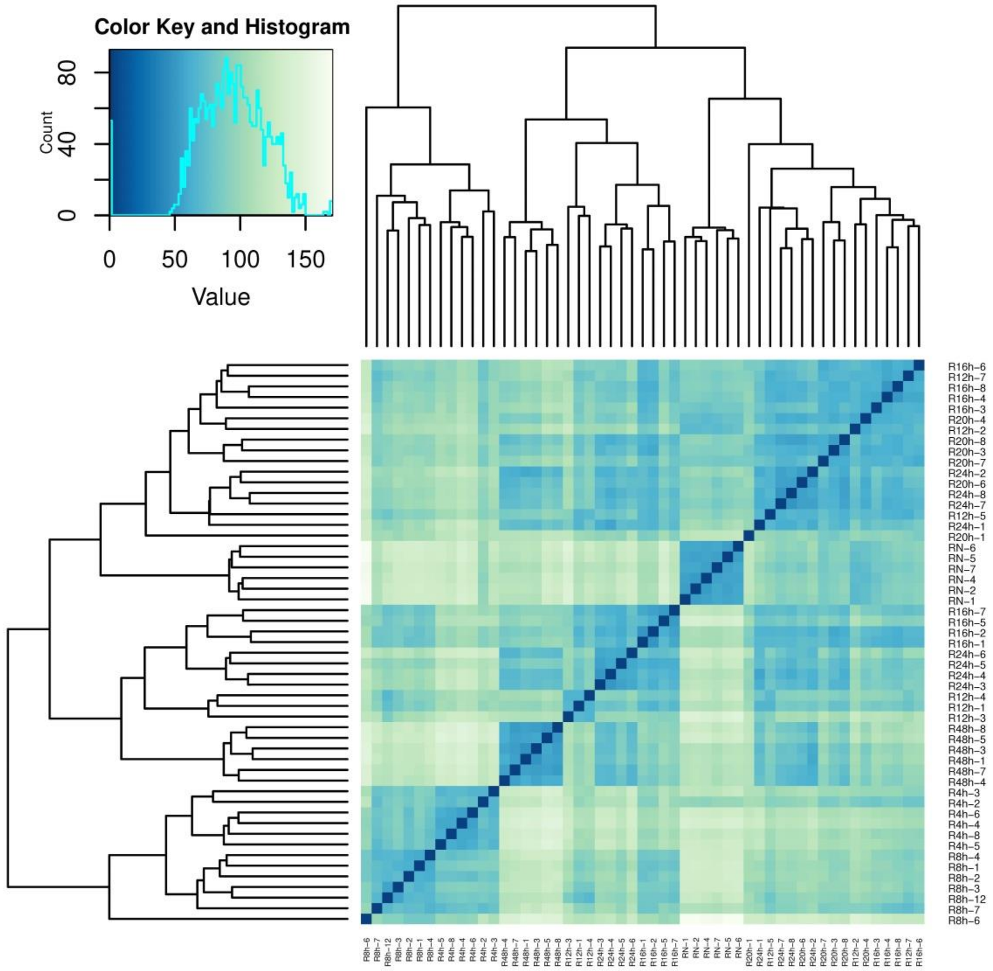

Fig.S4 The clustering relation heat map of all samples among experimental groups and control group. The samples almost clustered in six groups.

Supplementary Fig.5

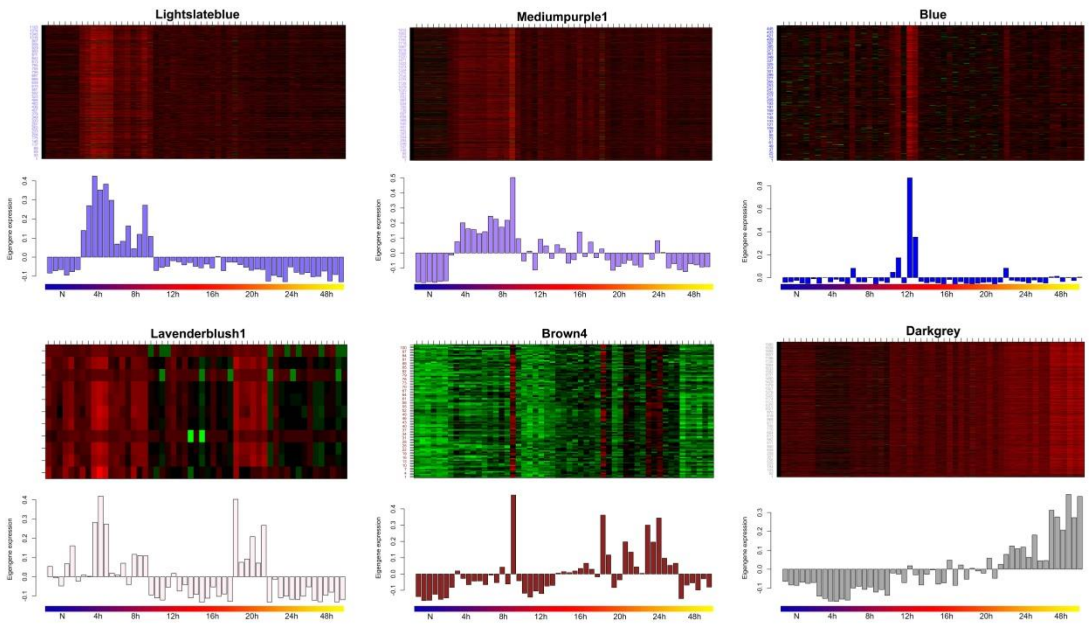

Fig.S5 Distribution of eigengene genes expression in each selected HCr module. The heat maps show the expression distribution of all genes in each HCr module; The different colors of the bar diagrams under the heat maps show the expression of eigengene in each HCr module during the whole time line in this study.

Supplementary Fig.6

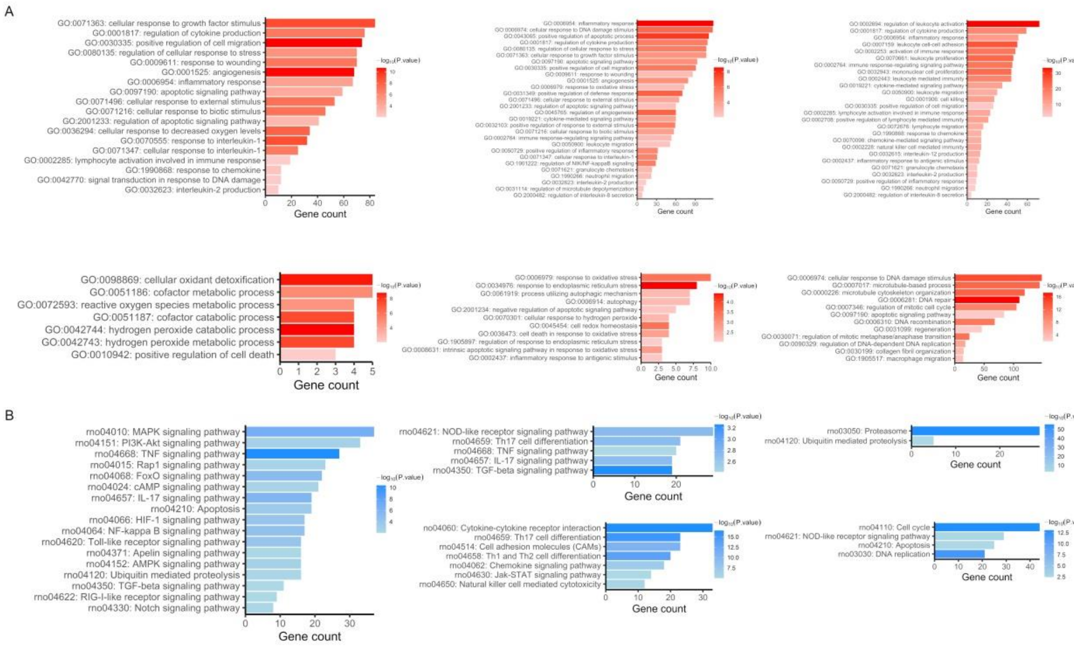

Fig.S6 Bar graphs of the distribution of GO and KEGG items in HCr modules in each time period after injury A The bioprocesses of GO terms in each HCr module were listed by the red bar graphs. The names and the GO id of the GO terms we interested were selected by p value listed on the left and ordered by the gene counts in each module. The color shade of the bar graph represents the size of p value. **B** The KEGG pathways in each HCr module were listed by the blue bar graphs. The names and the id of the KEGG pathways we interested were selected by p value listed on the left and ordered by the gene counts in each module. The color shade of the bar graph represents the size of p value.

Supplementary Fig.7

A

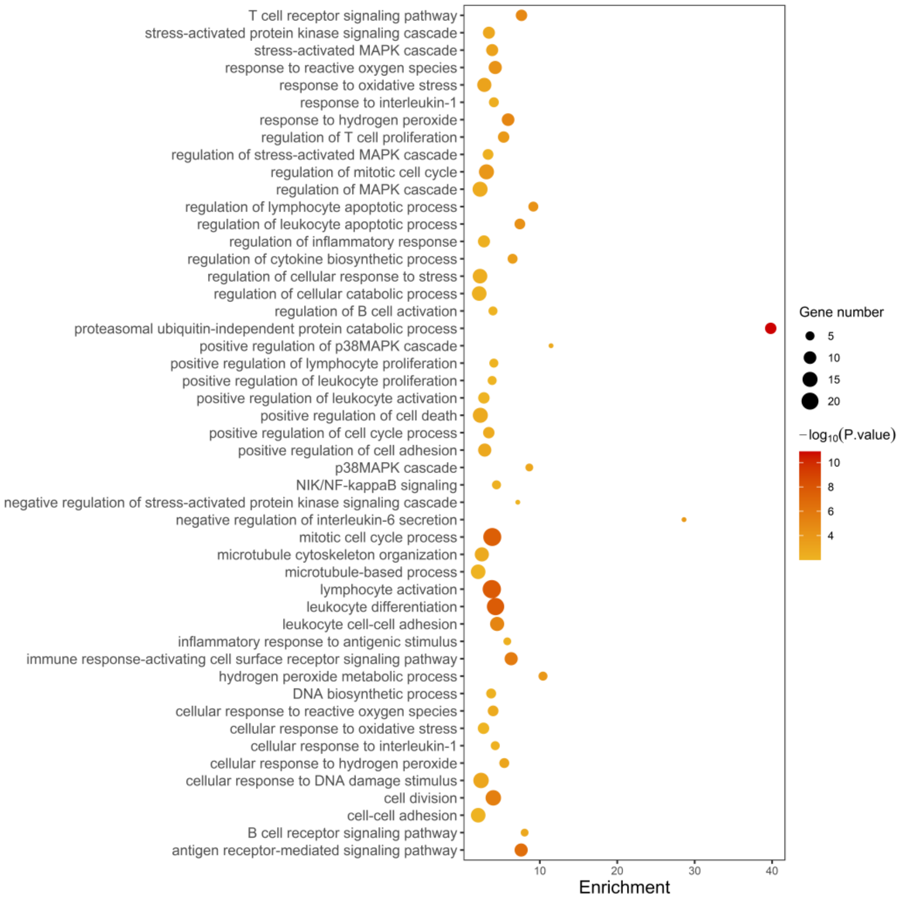

B

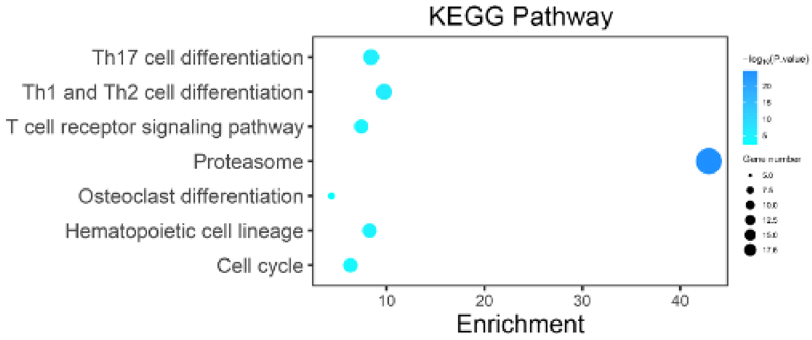

Fig.S7 The bubble diagram of GO terms and KEGG pathways in hub gene set. **A.** The names of GO terms (bioprocesses) in hub gene set (in total 161 genes) were listed on the top. The size of bubbles represent the numbers of genes in each GO terms and the color shade of bubbles represent the size of p value. The 50 interested

GO terms we selected by p value were distributed by enrichment of scores. **B.** KEGG pathway annotation of 161 hub genes (enrichment cutoff: $p < 0.05$). The size of bubbles represent the numbers of genes in each KEGG pathways and the color shade of bubbles represent the size of p value.

Supplementary Fig.8

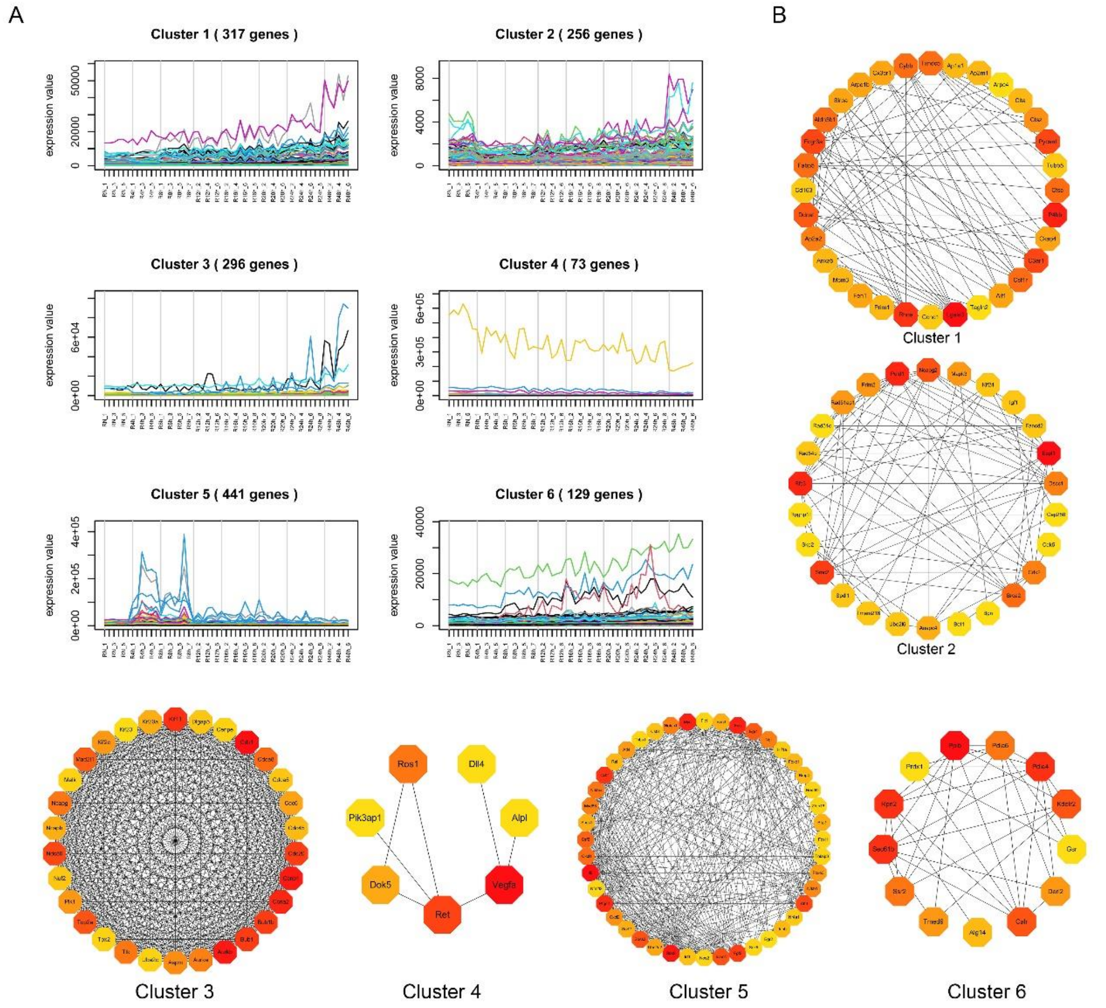

Fig.S8 The time dependent gene screening by maSigPro A Time dependent marker genes analysis and cluster visualization by R package masigpro(R-squared =0.7, k = 6) **B** The hub genes in PPI network analysis about the 3 clusters of maSigPro analysis and visualization by a plug-in cytohubba in cytoscape software. Here, we show the remaining four clusters.

Supplementary Fig.9

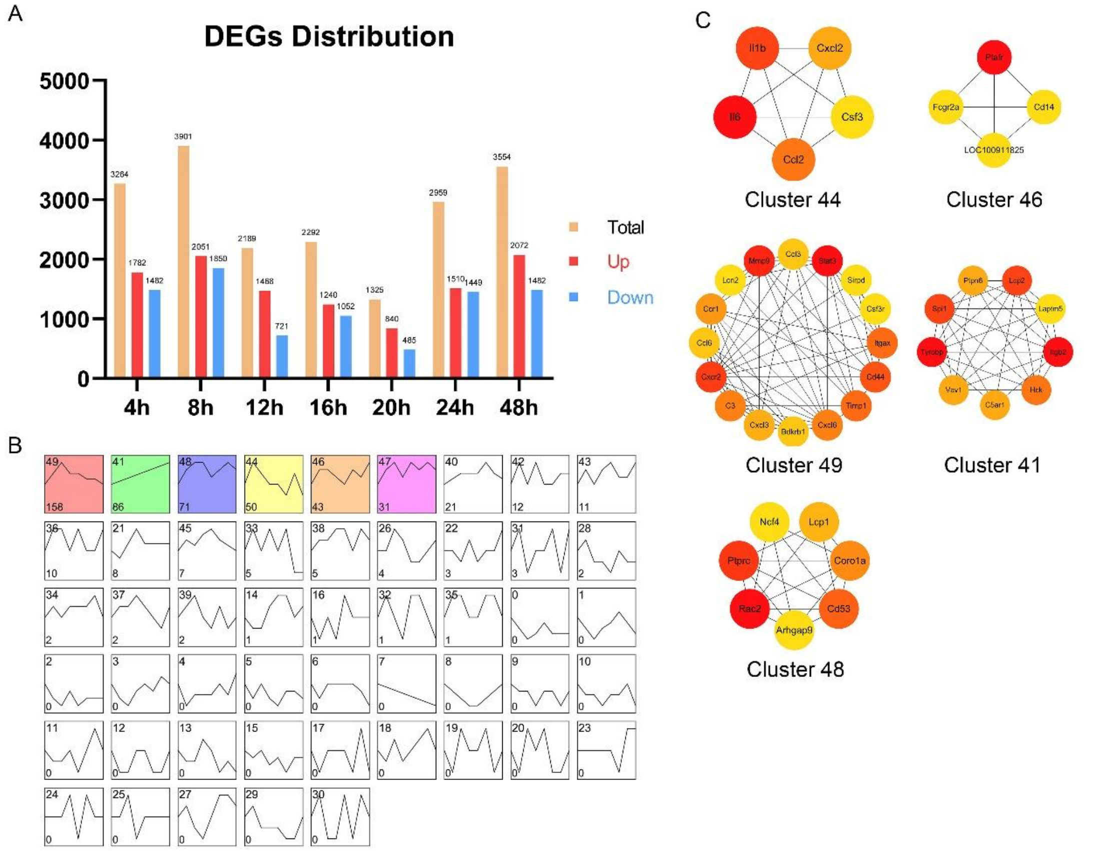

Fig.S9 DEGs distribution and time dependent gene screening by STEM **A** The bar graphs showed the numbers of up-regulated, down-regulated and total DEGs distribution in each time period after injury (｜$\log_2$FC｜≥1). **B** Short Time-series Expression Miner (STEM) analysis of the DEGs. The colored boxes represent significantly enriched profiles (p value < 0.001). In each colored box, the number on top left corner represent the name of the clusters and the number on left bottom represent the p value. The boxes were ordered by p value. **C** The hub genes in PPI network analysis about the 2 clusters of STEM analysis and visualization by a plug-in cytohubba in cytoscape software. Here, we show the remaining three clusters. The number of 47 box could not build the PPI network.

Supplementary Fig.10

A

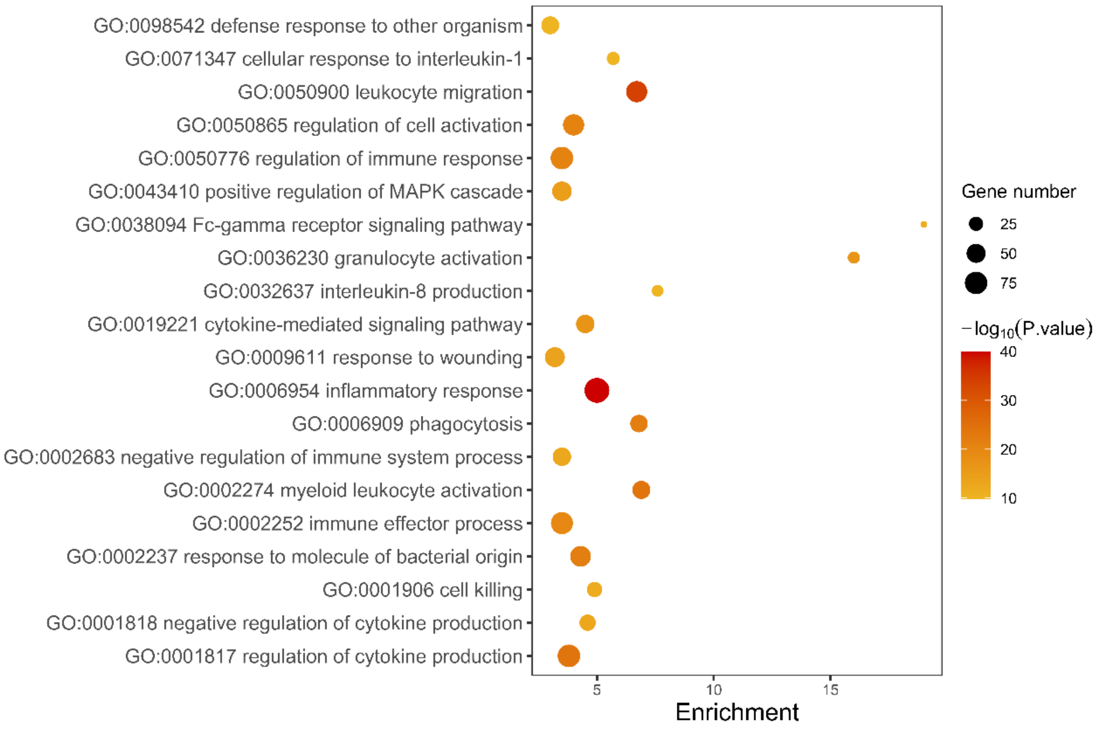

B

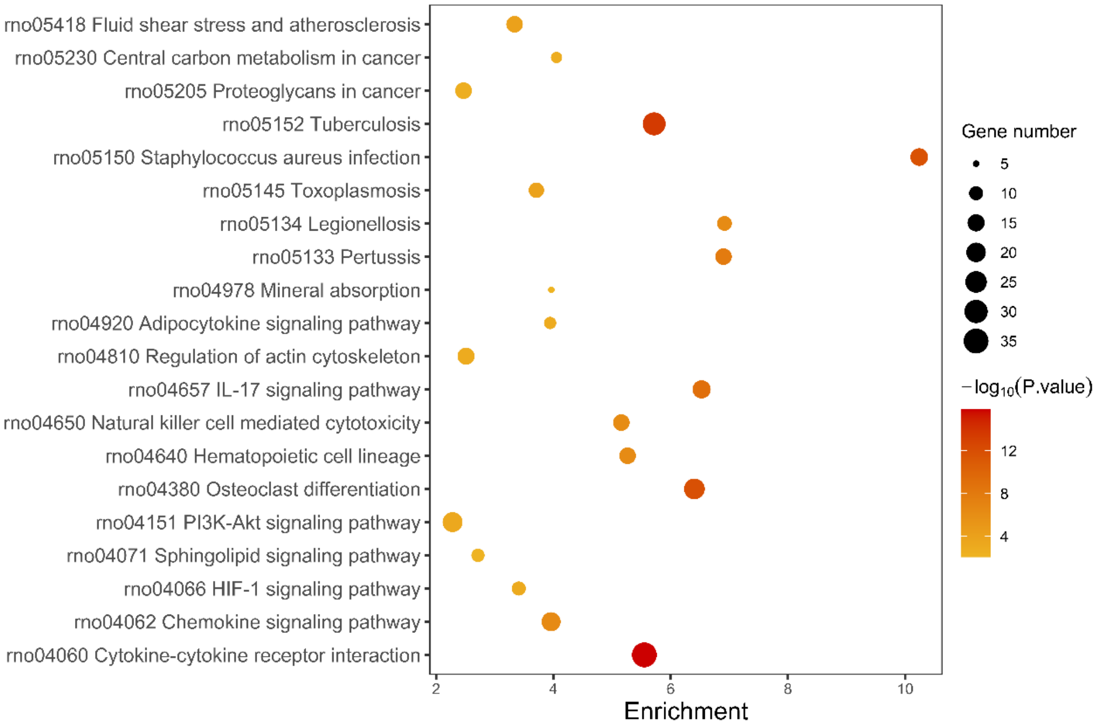

Fig.S10 The bubble diagram of GO terms and KEGG pathwats in common DE gene set. A. The names of GO terms (bioprocesses) in common DE gene set (in total

540 genes) were listed on the left. The size of bubbles represent the numbers of genes in each GO terms and the color shade of bubbles represent the size of p value. **B.** The names of KEGG pathways in common DE gene set were listed on the left. The size of bubbles represent the numbers of genes enriched in KEGG pathways and the color shade of bubbles represent the size of p value.

Supplementary Fig.11

A

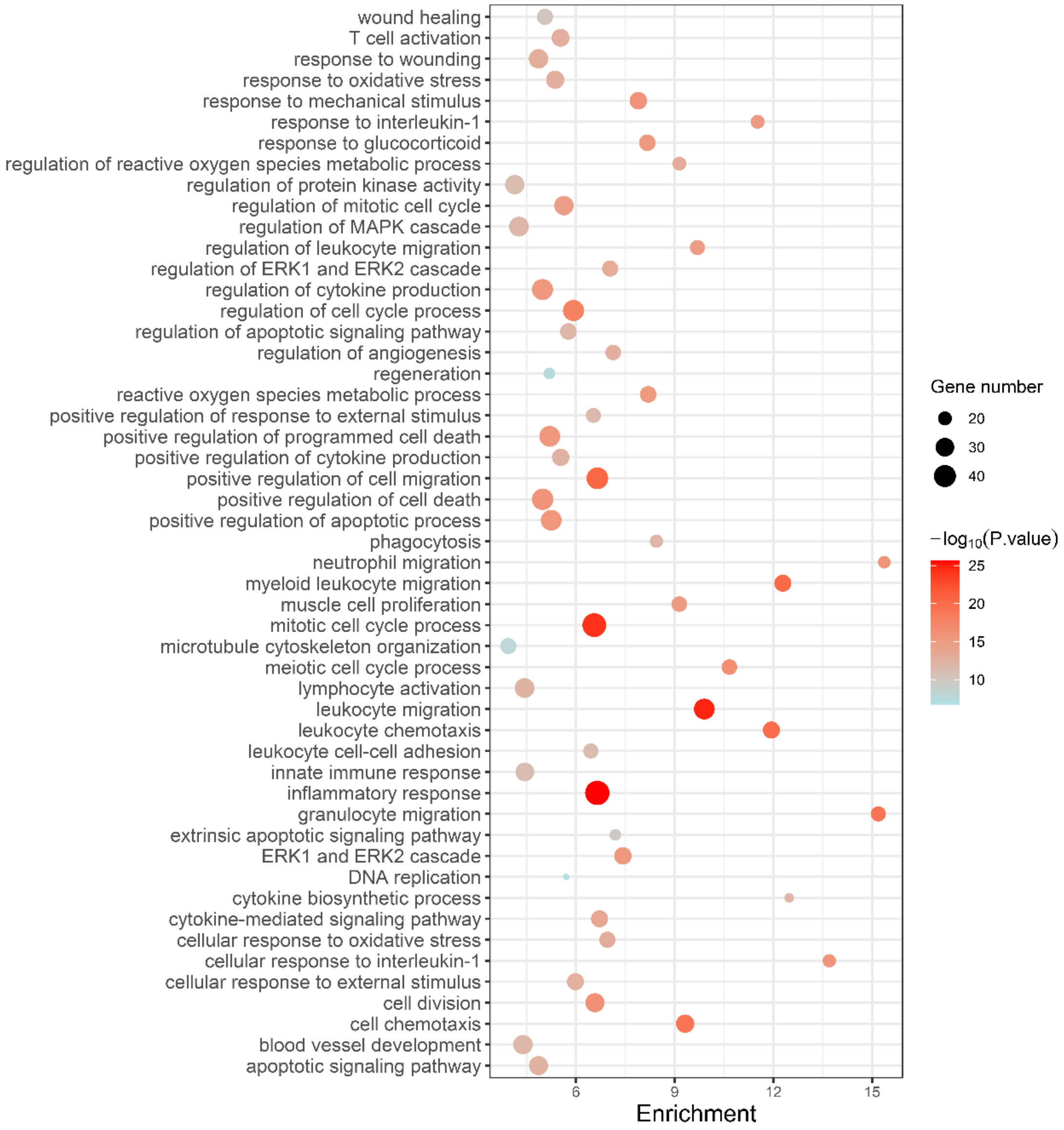

B

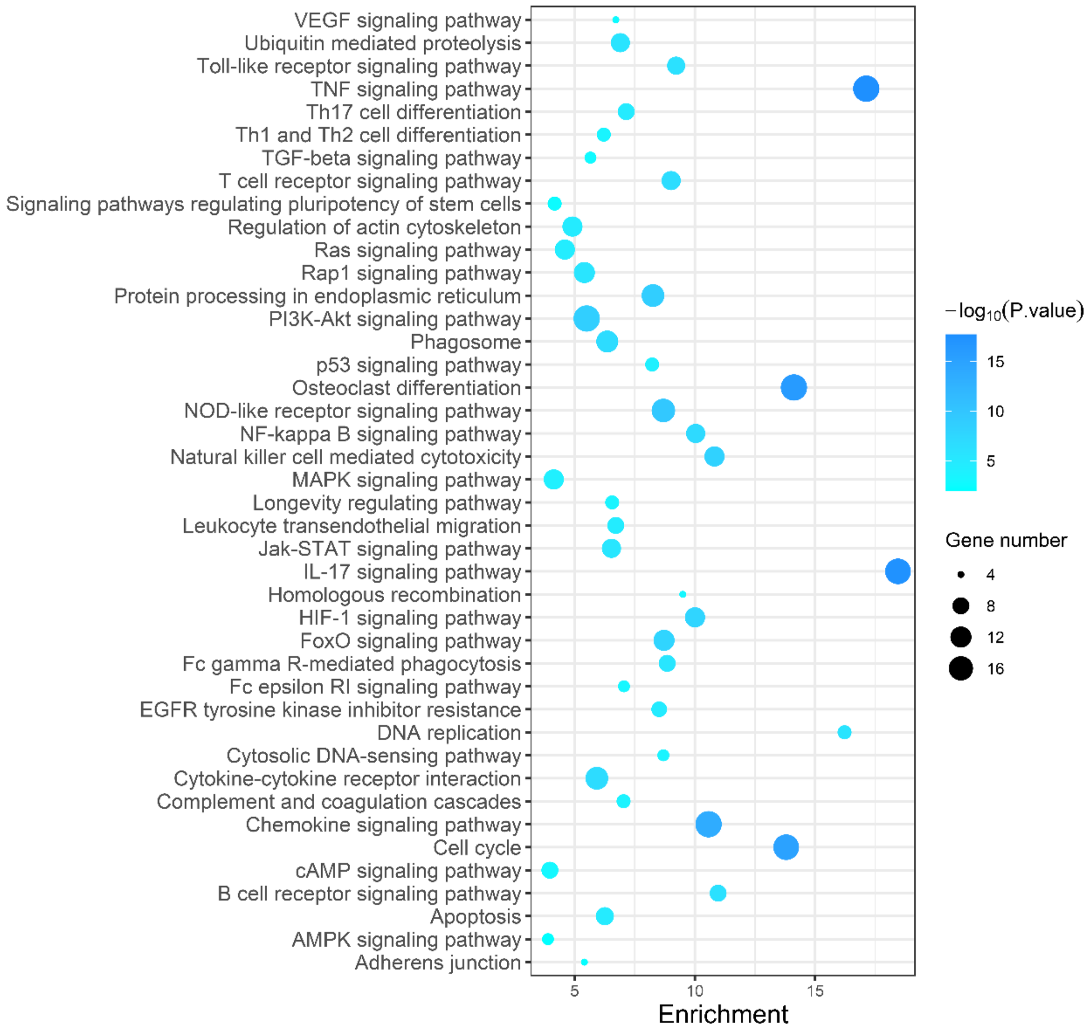

Fig.S11 The bubble diagram of GO terms and KEGG pathwats in 188 time-dependent genes. A. The names of GO terms (bioprocesses) in 188 time-dependent genes were listed on the left. The size of bubbles represent the numbers of genes in each GO terms and the color shade of bubbles represent the size of p value. **B.** The names of KEGG pathways in 188 time-dependent genes were listed on the left. The size of bubbles represent the numbers of genes enriched in KEGG pathways and the color shade of bubbles represent the size of p value.

Supplementary Tables:

Supplementary Tables are provided in a single Microsoft Excel file.

Supplementary Table 1 The DEseq standardization of gene expression profile

(The gene expression matrix at various time periods after skeletal muscle injury in rats was DESeq standardization of the original gene expression profile, 20662 genes)

Supplementary Table 2 The gene expression profiles for WGCNA analysis

(The gene expression profiles after selected by the threshold value from the original gene expression profiles for WGCNA analysis, 15901 genes)

Supplementary Table 3 The distribution of the time dependent gene sets in maSigPro clusters

(The distribution of the 6 time dependent gene clusters divided by maSigPro，1512 genes**)**

Supplementary Table 4 The common DEGs in 7 experiment groups for STEM analysis

(The counts of the common DEGs between the experimental groups at each time period after injury and the normal group for STEM analysis, 540 genes.)

Supplementary Table 5 The distribution of the time dependent gene sets in the selected clusters by STEM

(The distribution of the selected 6 time dependent gene clusters divided by STEM，439 genes)
